# A New Isoform of the Histone Demethylase JMJD2A/KDM4A Is Required for Skeletal Muscle Differentiation

**DOI:** 10.1371/journal.pgen.1001390

**Published:** 2011-06-02

**Authors:** Laure Verrier, Fabrice Escaffit, Catherine Chailleux, Didier Trouche, Marie Vandromme

**Affiliations:** 1 Université de Toulouse, UPS, LBCMCP, F-31062, Toulouse, France; 2 CNRS, LBCMCP, F-31062, Toulouse, France; The Jackson Laboratory, United States of America

## Abstract

In proliferating myoblasts, muscle specific genes are silenced by epigenetic modifications at their promoters, including histone H3K9 methylation. Derepression of the promoter of the gene encoding the myogenic factor myogenin (*Myog*) is key for initiation of muscle differentiation. The mechanism of H3K9 demethylation at the *Myog* promoter is unclear, however. Here, we identify an isoform of the histone demethylase JMJD2A/KDM4A that lacks the N-terminal demethylase domain (ΔN-JMJD2A). The amount of ΔN-JMJD2A increases during differentiation of C2C12 myoblasts into myotubes. Genome-wide expression profiling and exon-specific siRNA knockdown indicate that, in contrast to the full-length protein, ΔN-JMJD2A is necessary for myotube formation and muscle-specific gene expression. Moreover, ΔN-JMJD2A promotes MyoD-induced conversion of NIH3T3 cells into muscle cells. ChIP-on-chip analysis indicates that ΔN-JMJD2A binds to genes mainly involved in transcriptional control and that this binding is linked to gene activation. ΔN-JMJD2A is recruited to the *Myog* promoter at the onset of differentiation. This binding is essential to promote the demethylation of H3K9me2 and H3K9me3. We conclude that induction of the ΔN-JMJD2A isoform is crucial for muscle differentiation: by directing the removal of repressive chromatin marks at the *Myog* promoter, it promotes transcriptional activation of the *Myog* gene and thus contributes to initiation of muscle-specific gene expression.

## Author Summary

Gene expression is regulated in part by so-called “epigenetic” mechanisms—modifications of the DNA itself or of proteins associated with DNA (such as histones). We have studied an enzyme that affects these histone modifications—the histone demethylase JMJD2A—and its role in muscle differentiation. Muscle differentiation is tightly controlled during the development of the organism to ensure that muscle forms at the right place and at the right time. Muscle precursor cells are prevented from differentiating by silencing of a “master gene” of muscle differentiation called *Myog*. This silencing is brought about by specific epigenetic modifications of the *Myog* gene that are removed when the precursor cell receives a signal to differentiate. Here, we have discovered a new isoform of JMJD2A that is upregulated during muscle differentiation and that is required for activation of *Myog*. Binding of this JMJD2A isoform to the *Myog* gene is probably a crucial step in inducing muscle differentiation. Our findings thus uncover the existence of a second isoform of the histone demethylase JMJD2A important for muscle differentiation and suggest that the balance between JMJD2A isoforms is important for controlling the fate of muscle precursor cells.

## Introduction

In eukaryotes, the genome is organized into a highly ordered structure called chromatin, composed of DNA and histone proteins. Chromatin architecture is dynamic and regulated in part by enzymes that mediate covalent post-translational modifications of the N-terminal tails of histones. These so-called epigenetic modifications of histones control the expression of the genes they are associated with, thus they are crucial in determining cell fate decisions.

Enzymes that remove methyl groups from lysine residues in histones (histone lysine demethylases, KDMs) were discovered only recently and are highly specific for particular lysine residues, similarly to histone lysine (K) MethylTransferases (KMT) [1]. The first discovered demethylase, LSD1, is a flavin-monoamine oxidase that demethylates mono- or dimethylated lysine residues 4 and 9 in histone H3 (H3K4 and H3K9) [2]. The Jumonji domain-containing demethylases catalyze protein demethylation through a hydroxylation reaction requiring iron and 2-oxoglutarate as co-factors. The JMJD2/KDM4 subfamily (JMJD2A, B, C and D) specifically demethylates H3K9 and H3K36 either di- or tri-methylated [3]–[7].

Few regulatory functions have been described for the JMJD2 family. JMJD2C was described as a co-activator of androgen responsive genes in prostate cancer cells and of the Nanog gene in ES cells [8]–[11]. By contrast, mainly co-repressor activities have been reported for JMJD2A: as a component of the nuclear receptor co-repressor (N-CoR) complex involved in the repression of the *ASCL2* gene [12], a function that requires its demethylase activity [6], and associated with histone deacetylases and the retinoblastoma protein Rb to repress E2F-regulated promoters (in this case, the role of its demethylase activity was not investigated) [13]. JMJD2A was also suggested to be a co-activator of androgen receptor-dependent gene transcription [14], but this is controversial since another group claimed that this function is specific to JMJD2C [8].

During skeletal muscle differentiation, signaling to chromatin is important to drive the correct temporal expression of muscle-specific genes [15]. During this process, myoblasts cease proliferating and fuse into multinucleated myotubes; at the same time, muscle specific genes begin to be transcribed and cell cycle-associated genes are repressed. The b-HLH family of myogenic transcription factors (including MyoD, Myf5, myogenin and MRF4) and the MEF2 factors play key roles in controlling muscle-specific gene expression. In response to appropriate differentiation signals, MyoD, which is expressed in myoblasts, turns on the differentiation program by first activating expression of the gene encoding myogenin (*Myog*). Then, in cooperation with myogenin, it induces the expression of many other muscle specific genes [16]. MyoD and MEF2 factors act in concert with histone acetyltransferases and deacetylases [17] to influence muscle gene transcription by modifying the architecture of chromatin at specific loci.

Whereas the role of histone acetylation in the control of muscle-specific gene expression has been studied extensively (reviewed in [17]), less is known about the role of histone methylation in this process. The Polycomb group protein Ezh2, a methyltransferase, is involved in inhibiting muscle differentiation by methylating H3K27 at muscle-specific genes regulatory elements in myoblasts [18]. The role of Ezh2 is counteracted by the histone demethylase UTX, which demethylates H3K27 to participate in the activation of muscle-specific loci during differentiation [19]. Ash2L methyltransferase-containing complexes also contribute to this activation by methylating H3K4 [20]. In addition to H3K27, H3K9 methylation also participates in *Myog* repression, ensuring that cells do not differentiate prematurely. The *Myog* promoter is hypermethylated on H3K9 in myoblasts and demethylated in myotubes concomitant with transcriptional activation of the *Myog* gene [21]. The histone methyltransferase Suv39h1 is responsible for the establishment of this repressive epigenetic mark in myoblasts, and is targeted to the *Myog* promoter by interacting with MyoD [22], [23]. The mechanism leading to H3K9 demethylation of the *Myog* promoter at the onset of differentiation has not been studied extensively, however.

Here, we identify a new isoform of JMJD2A in which the N-terminal demethylase domain has been deleted (ΔN-JMJD2A). This isoform is upregulated at the onset of myoblast differentiation into myotubes and is essential for this process. By using a genome-wide approach, we show that ΔN-JMJD2A acts as a transcriptional co-activator for many genes induced specifically during skeletal muscle differentiation. Furthermore, we demonstrate that ΔN-JMJD2A binds directly to the *Myog* promoter to regulate H3K9 methylation, thus allowing *Myog* expression and promoting myotube differentiation.

## Results

### A short isoform of JMJD2A is upregulated during muscle differentiation

**Figure 1:**
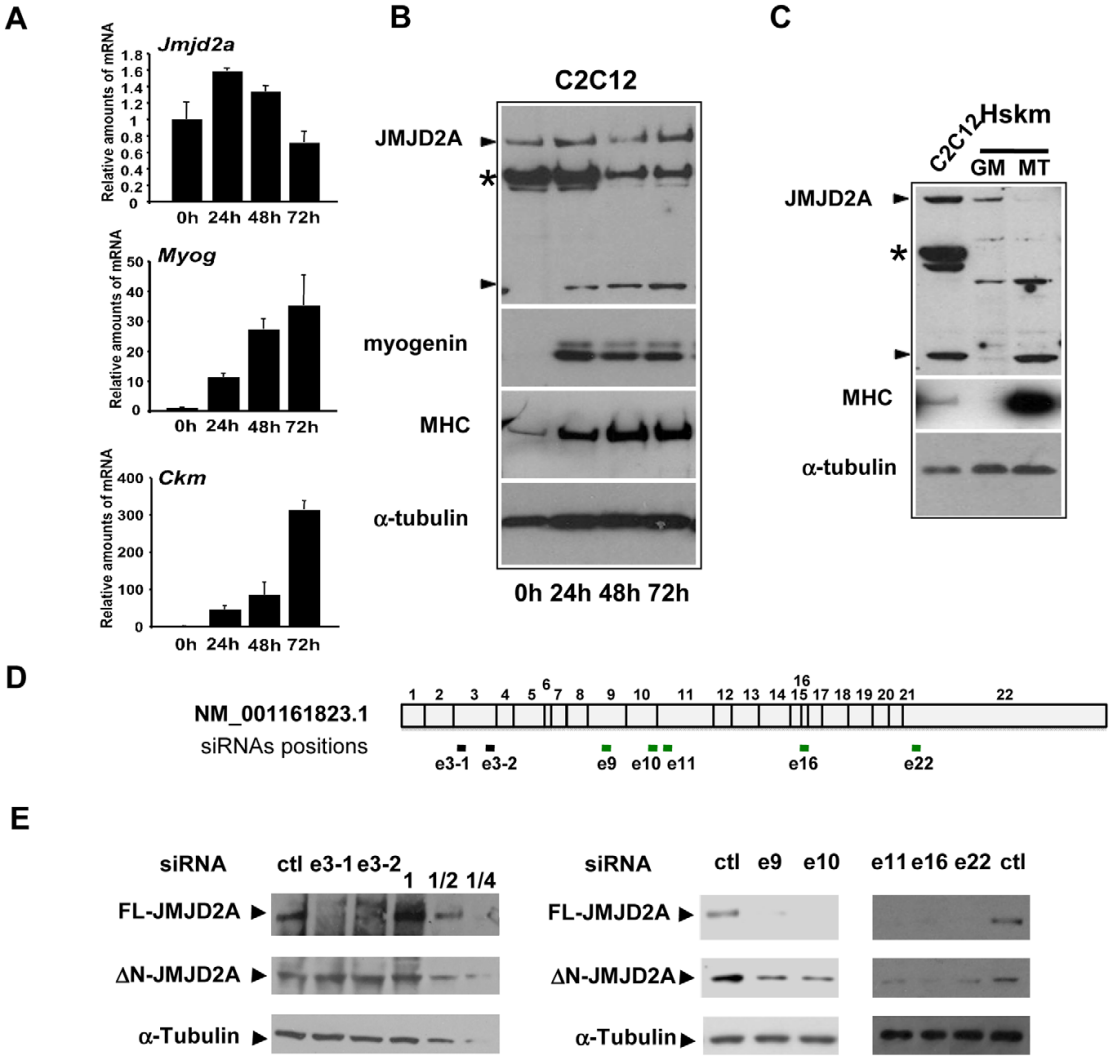
Expression of *JMJD2A* during muscle differentiation. (A) The expression of *JMJD2A*, *Myog*, *Ckm* and *gapdh* mRNA was measured by RT-qPCR of RNA from C2C12 cells kept in growth medium (0 h) or shifted to differentiation medium for 24, 48 or 72 hours. mRNA levels were standardized to *gapdh* mRNA levels, and calculated relative to 1 in proliferating cells. (B) Western blots of total protein extracts from C2C12 cells before (0 h) or after 24, 48 or 72 hours of differentiation analyzed for the presence of JMJD2A (α-Cter antibody), myogenin, myosin heavy chain (MHC) and α-tubulin (as a control for equal loading). The asterisk (*) indicates a non-specific band (as indicated by subsequent analysis using siRNAs and through the use of other antibodies; data not shown). JMJD2A-specific bands are indicated by arrowheads. Note that we show two different exposures of this western blot (C)Western blots of total protein extracts from human skeletal myoblasts (Hskm) in proliferating (GM) or differentiated conditions (MT), and from differentiating C2C12 cells (left lane) analyzed for expression of JMJD2A (α-Cter antibody), myosin heavy chain (MHC) and α-tubulin. JMJD2A-specific bands are indicated by arrowheads. The asterisk (*) indicates a non-specific band. (D) Schematic representation of *JMJD2A m*RNA(NM_001161823.1) indicating the positions of hybridisation of the siRNAs used in this study. The black bars indicate the siRNAs that knockdown only full-length JMJD2A protein expression whereas the green bars indicate the siRNAs that knockdown expression of both the full-length and the short isoform. The siRNAs are named according to the exon they target and the position in the exon (e.g. sie3-1 for the most upstream siRNA targeting exon 3). (E) C2C12 cells were transfected with siRNAs targeting both isoforms of JMJD2A (e9, e10, e16 and e22), only full-length JMJD2A (e3-1 and e3-2) or control siRNA (ctl). After 24 hours of differentiation, western blots of total protein extracts were analysed for JMJD2A (α-Cter antibody) and α-tubulin. The panels on the left include a serial dilution (1, ½ and ¼) of extracts from cells transfected with the control siRNA.

To investigate the role of the histone demethylase JMJD2A in muscle differentiation, we first characterized expression of JMJD2A mRNA in a mouse myoblast cell line, C2C12, which has been used extensively as a model of skeletal muscle differentiation. C2C12 cells differentiated into myotubes expressing muscle specific genes within three days after addition of differentiating medium, as demonstrated by the strong induction of mRNAs from the *Myog* and muscle creatine kinase (*Ckm*) genes (Figure 1A). By contrast, JMJD2A mRNA, as quantified by RT-qPCR using primers located in its 3′end, was expressed at relatively constant levels during this time (Figure 1A). As shown in Figure 1A (upper panel), JMJD2A mRNA, quantified by RT-qPCR using primers located in its 3′end, is expressed at relatively constant levels during the course of C2C12 cells differentiation. We also analyzed JMJD2A protein levels by western blotting the cells with an antibody directed against the C-terminal part of JMJD2A (α-Cter-JMJD2A). In addition to the full-length protein, whose levels remained constant, we noticed a smaller isoform of about 60 kDa in differentiating C2C12 cells (Figure 1B). In primary human satellite cells, differentiation was also accompanied by the appearance of a polypeptide of very similar apparent molecular weight to that observed in C2C12 cells (Figure 1C). These data suggest the existence of a new isoform of JMJD2A whose expression increases during muscle terminal differentiation. Note that this isoform can also be detected in proliferating C2C12 cells when the blots are exposed for longer times (data not shown). To prove that this isoform was derived from JMJD2A, and to gain insights into the mRNA producing it, we transfected differentiating C2C12 cells with exon-specific siRNAs against various parts of JMJD2A mRNA (Figure 1D). As expected, all siRNAs interfered with expression of the full-length protein (Figure 1E). Strikingly, whereas the siRNAs against exons 9, 10, 11, 16 and 22 of JMJD2A, also decreased expression of the short isoform, those against exon 3 did not (Figure 1E), This suggests that the small isoform of JMJD2A is produced from a mRNA derived from the JMJD2A gene but lacking the 5′ end of the full-length JMJD2A mRNA.

### The short isoform is produced from an mRNA that initiates within the coding region of the *JMJD2A* gene

**Figure 2:**
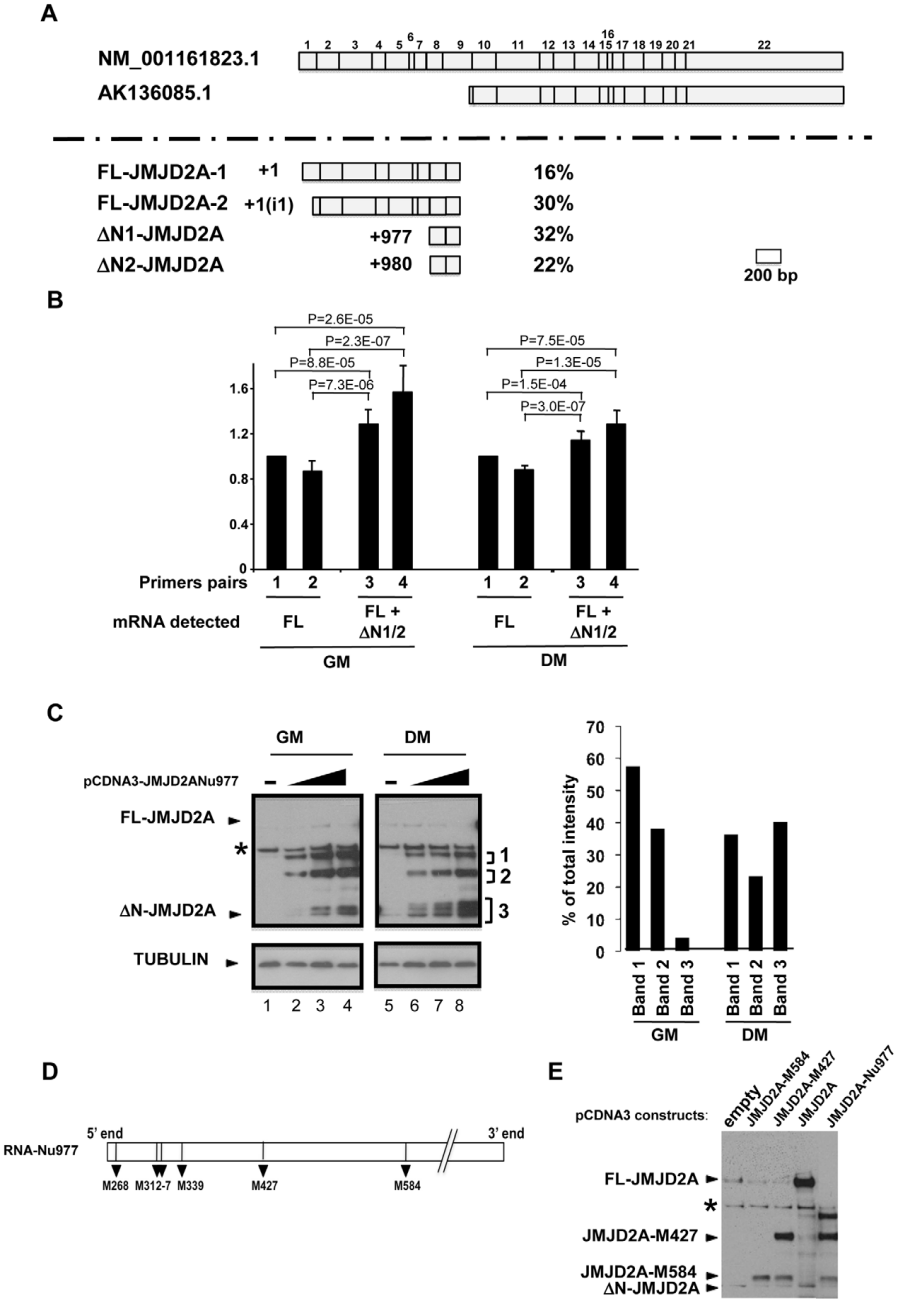
Characterization of the small isoform of JMJD2A. (A) 5′ RACE was performed on mRNA extracted from differentiated C2C12 cells. The two JMJD2A mRNAs described in Genbank are illustrated in the upper panel, with the exons labelled from 1 to 22. The various 5′ends identified by 5′ RACE are shown in the lower panel. The start positions relative to the first nucleotide of the described full-length mRNA (NM_001161823.1) are indicated on the left, and the percentage of each mRNA obtained in our experiments on the right. The +1(i1) indicates mRNAs initiating within intron 1 of *JMJD2A* (FL for full-length; ΔN for N-terminal deleted). (B) The amount of *JMJD2A* mRNA in total RNA prepared from C2C12 cells kept in growth medium (GM) or in differentiation medium for 72 hours (DM) was quantified by qPCR (relative to a standard curve with known amounts of *JMJD2A* gene) by using primer pairs that amplified *FL-JMJD2A* mRNA only (pairs 1 and 2) or *FL-JMJD2A*, *ΔN1-JMJD2A* and *ΔN2-JMJD2A* mRNAs (pairs 2 and 3). Quantification was performed twice in triplicate on four independent mRNA preparations from proliferating cells and differentiated myotubes. Data were standardised relative to 1 for primer pairs 1 (corresponding to about 10^5^ molecules of *JMJD2A* cDNA per µg of cDNA); statistical analyses of the observed differences are indicated (p values, student T test). (C) The expression of JMJD2A was analysed by western blotting of growing C2C12 myoblasts (GM) or after 24 hours of differentiation (DM) using the antibody recognizing both isoforms of JMJD2A (α-Cter antibody); α-tubulin was blotted as a loading control. Cells were transfected with increasing doses (0.05, 0.1 or 0.2 µg for myoblasts, lanes 2–4; 0.2, 0.4 or 1 µg for differentiating cells, lanes 6–8) of a vector expressing the short *JMJD2A* mRNA beginning at nucleotide 977 of the full-length mRNA (pCDNA3-JMJD2A-Nu977) or with the empty vector (lanes 1 and 5). The intensities of the three bands (1, 2 and 3) produced from the pCDNA3-JMJD2ANu977 construct were quantified using ImageJ software (right panel). The data are represented as the percentage of each band relative to the total intensity of the three bands, calculated for the first dose of Nu977 expression vector (to remain in the linear range of fluorography). (D) Schematic representation of the *JMJD2A-Nu977* mRNA with potential translation initiation codons. (E) Growing C2C12 cells were transfected with 0.1 µg of vector expressing FL-JMJD2A or N-terminal deletion mutants (JMJD2A-M427, JMJD2A-M584). The expression of JMJD2A was analysed by western blotting using the antibody recognizing both isoforms of JMJD2A (α-Cter antibody).

The Genbank database contains a description of a small mRNA (AK136085.1) whose transcription starts inside the coding sequence of the for *JMJD2A* gene and which is identical to the full-length mRNA sequence from the end of exon 9 to the 3′ end of JMJD2A variant 1 (NM_001161823.1; see Figure S1 for an alignment). The siRNA that targeted the beginning of exon 9 of *JMJD2A* inhibited production of the small isoform described in Figure 1, however, indicating that the mRNA that encodes the small isoform differs from AK136085.1. To identify the 5′end of this mRNA, we performed rapid amplification of 5′ complementary DNA ends (5′RACE) using total mRNA isolated from differentiated C2C12 cells. We designed the primers according to the effects observed on the short isoform expression using exon-specific siRNAs (see Figure 1D and 1E) and used them to isolate 50 clones containing parts of JMJD2A mRNA, which we then sequenced (Figure 2A). Among these, eight clones were identical to the 5′ end of the full-length sequence described in Genbank (NM_001161823.1). The transcription start site (TSS) of NM_001161823.1 was designated as +1 relative to this sequence. Fifteen clones started downstream of this TSS at a position located in the first intron of *JMJD2A*. Since the ATG corresponding to the full-length protein is present in exon 2, this latter mRNA is also likely to produce the full-length JMJD2A protein. Twenty seven clones initiated inside the coding region at the 5′ end of exon 8, of which 16 had a TSS at +977 and 11 at +980. Thus, a significant proportion of JMJD2A mRNAs initiate at the beginning of exon 8 in differentiating C2C12 cells (see Figure S1 for alignment of sequences).

To quantify the isoforms produced from the *JMJD2A* gene, we analysed their expression in proliferating cells and in differentiating cells by qPCR with primers located immediately upstream of nucleotide +977 (Nu977, primer pairs 1 and 2) or immediately downstream of Nu977 (primer pairs 3 and 4; Figure 2B). We found that the signal observed with primer pairs 3 and 4 was 20–30% higher than with primer pairs 1 and 2 in both proliferating and differentiated cells, indicating that ΔN1-JMJD2A and ΔN2-JMJD2A mRNAs are expressed at about 20% of the amount of the full-length mRNAs. In addition, the expression of these mRNAs slightly decreased during differentiation relative to the full-length (FL)-JMJD2A mRNA (Figure 2B).

To test if the small mRNA (ΔN1/2-JMJD2A) could encode the shorter of the two proteins we observed above (see Figure 1), we cloned the *JMJD2A* cDNA starting at nucleotide +977 and expressed it in proliferating and differentiating C2C12 cells. In cells kept in growth medium (GM) or shifted into differentiation medium for 24 h (DM), overexpression of this cDNA under the control of the CMV promoter led to production of various polypeptides with apparent molecular weights of 115, 85 and around 60 kDa (denoted 1–3 respectively, in Figure 2C). The 60 kDa polypeptides comprised two bands in proliferating myoblasts and three in differentiating cells, the fastest of these bands co-migrated exactly with the endogenous JMJD2A short isoform (seen in lane 5). These 60 kDa polypeptides were more abundant in differentiating cells; they represent about 40% of the total in differentiating cells compared to only 5% in proliferating myoblasts (Figure 2C, right). This suggests that the JMJD2A mRNA that initiates at nucleotide 977 directs production of a protein corresponding to the endogenous JMJD2A small isoform preferentially in differentiating cells.

Several potential initiation codons (ATG) are present in the Nu977 mRNA sequence (Figure 2). To characterize further the small isoform of JMJD2A, we constructed deletion mutants of JMJD2A and expressed them in C2C12 cells. The constructs were named according to their first methionines: pCDNA3-JMJD2A-M317 (data not shown), pCDNA3-JMJD2A-M427 and pCDNA3-JMJD2A-M584. A protein initiating at Met584 (JMJD2A-M584) migrated close to the endogenous small isoform (Figure 2E). Taken together with the data obtained by using isoform-specific siRNAs (Figure 1), these findings indicate that the small isoform is produced from an mRNA initiating within the *JMJD2A* gene. This shorter isoform lacks the N-terminal sequences of JMJD2A and thus lacks the demethylase domain; hereafter we call this isoform ΔN-JMJD2A.

### ΔN-JMJD2A is required for C2C12 cell differentiation

**Figure 3:**
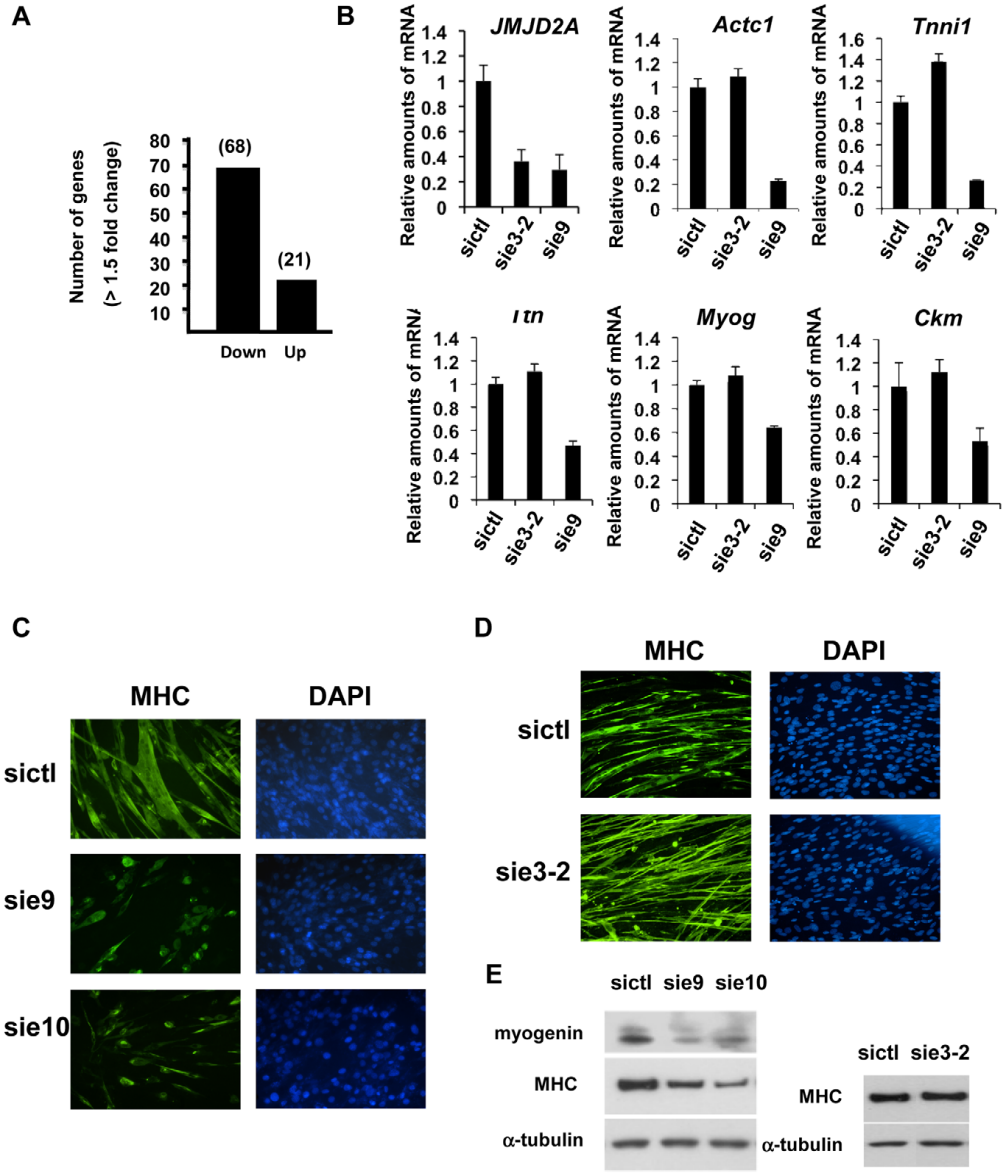
ΔN-JMJD2A is important for muscle differentiation and myotube formation. (A) Gene expression profiles of C2C12 cells treated with siRNAs targeting both isoforms (sie9 or sie10, directed against exon 9 and 10 respectively) or control siRNA and differentiated for 36 h, were analysed using Affymetrix expression arrays as described in Materials and Methods. The numbers of genes that are found down- or up-regulated in cells transfected with siRNA targeting both isoforms compared to control (fold change >1.5 when compared to the control) are indicated. (B) Validation of the microarray results by RT-qPCR analysis of mRNA expression of various differentiation-related genes shown in Table 1, as well as *FL-JMJD2A* (using primers located in the 5′ end). C2C12 cells were transfected with an siRNA targeting both isoforms of JMJD2A (sie9), a siRNA targeting only FL-JMJD2A (sie3-2, directed against exon3) or a control siRNA (sictl) and then induced to differentiate for 36 hours. mRNA levels were standardized according to *gapdh* mRNA levels, and calculated relative to 1 for cells transfected by the control siRNA. (C) C2C12 cells were transfected with siRNAs targeting both isoforms of JMJD2A (sie9 and sie10) or control siRNA (sictl). After 72 hours of differentiation, cells were stained for immunofluorescence microscopy by using an antibody against myosin heavy chain (MHC) and DAPI. (D) As in C, but C2C12 cells were transfected with siRNA targeting FL-JMJD2A only (sie3-2) or control siRNA (sictl). (E) Western blots of total protein extracts prepared from C2C12 cells treated as in C and D, showing myogenin, myosin heavy chain (MHC) and α-tubulin.

To investigate the role of ΔN-JMJD2A, we performed genome-wide expression profiling of C2C12 cells following transfection with siRNAs against both isoforms of JMJD2A. (Since the mRNA encoding ΔN-JMJD2A initiates in an exon, it is impossible to design specific siRNAs for this isoform). We transfected C2C12 cells with siRNAs that target exon 9 or exon 10 (sie9 and sie10) and maintained them in proliferation medium for 24 h before shifting them to differentiation medium for 36 hours. (Importantly, these siRNA did not affect JMJD2B and JMJD2C mRNAs; Figure S2). Total mRNA was hybridized onto the GeneChip Mouse Gene 1.0 ST Affymetrix microarray. Genes were considered as deregulated upon JMJD2A knockdown, as compared to a control siRNA (p-value <0.05), when the calculated average fold change was greater than 1.5 in three independent experiments with two independent siRNAs. We found that the expression of 89 genes was affected by JMJD2A knockdown, of which 68 were downregulated (Figure 3A) (See Table S1 for the list of JMJD2A-regulated genes). Gene ontology (GO) analysis using DAVID software indicated that a large proportion of these genes (21 out of 68) were either muscle specific or associated with muscle development and differentiation (see Table 1 for the list of muscle relevant genes). Most of these genes (16 out of 21) were up regulated upon C2C12 differentiation (Blais *et al*., 2005). Interestingly, expression of these genes was unchanged in similar experiments performed with siRNAs that decrease only the full-length protein (the changes affecting all these mRNA were classified as non significant, whereas JMJD2A mRNA was found decreased by 3.4 fold, data not shown), suggesting that they are specifically regulated by ΔN-JMJD2A. To confirm these findings, we analyzed the expression of several of the affected genes (*Actc1*, *Tnni1*, *Ttn*, *Myog*, *Ckm*) by reverse transcription followed by qPCR. We found that a siRNA against both forms of JMJD2A (sie9) strongly decreased expression of all of these genes, whereas a siRNA against the full-length isoform only (sie3-2) had no effect, although they both decreased similarly the expression of full-length JMJD2A (Figure 3B). These data confirm the DNA microarray analysis and indicate that these muscle-specific genes are specifically activated, either directly or indirectly, by ΔN-JMJD2A.

**Table 1:** Genes associated with muscle differentiation and development (gene ontology analysis; Figure 2B) that were down-regulated in cells transfected by siRNA targeting both isoforms of JMJD2A.

**Tnnc2**	troponin C2, fast	−4.8898516
**Myl1**	myosin, light polypeptide 1	−4.3695893
**Ckm**	creatine kinase, muscle	−3.976595
**Myog**	myogenin	−3.6869516
**Tnni1**	troponin I, skeletal, slow 1	−3.3599658
**Tnni2**	troponin I, skeletal, fast 2	−3.2088106
**Tnnt3**	troponin T3, skeletal, fast	−3.0840454
**Tnnt2**	troponin T2, cardiac	−2.9699507
**Erbb3**	v-erb-b2 erythroblastic leukemia viral oncogene homolog 3 (avian)	−2.8317823
**Acta1**	actin, alpha 1, skeletal muscle	−2.6396954
**Unc4b**	unc-45 homolog B (C. elegans)	−2.3936517
**Mef2c**	myocyte enhancer factor 2C	−2.2935529
**Actc1**	actin, alpha, cardiac	−1.9588187
**Ckb**	creatine kinase, brain	−1.915279
**Tmod1**	tropomodulin 1	−1.8805943
**Ttn**	titin	−1.7811661
**Ednra**	endothelin receptor type A	−1.7055933
**Tpm3**	tropomyosin 3, gamma	−1.6838151
**Chrnd**	cholinergic receptor, nicotinic, delta polypeptide	−1.6777709
**Dok7**	docking protein 7	−1.6551543
**Akt2**	thymoma viral proto-oncogene 2 | similar to serine/threonine kinase	−1.5684415
**Snf1lk**	SNF1-like kinase	−1.5214148

The fold change in gene expression is indicated for cells treated with siRNA against JMJD2A compared to cells treated with control siRNA.

To test whether ΔN-JMJD2A plays a role in muscle differentiation, we knocked down both isoforms in proliferating C2C12 cells and examined their ability to undergo differentiation. Proliferating C2C12 cells were electroporated with the siRNAs against both isoforms and kept in growth medium for 24 hours before incubating them in differentiation medium for 24 hours. The cells were then transfected again with the same siRNA, in order to maintain the knockdown, and kept in differentiation medium for a further 48 hours to allow full differentiation. Differentiation was assayed by expression of myosin heavy chain (MHC) by immunofluorescence microscopy. MHC expression and myotube formation were drastically impaired in cells transfected with the siRNAs that interfered with both isoforms (Figure 3C), but not by a siRNA that interfered with only FL-JMJD2A or by the control siRNA (Figure 3D). Western blotting showed that, in addition to MHC, myogenin expression was also impaired specifically by the siRNA targeting both isoforms (Figure 3E). These findings agree with those obtained from analysis of the expression of these differentiation markers by RT-qPCR (Figure 3B). Together, these data indicate that ΔN-JMJD2A is required for muscle differentiation at a step before myogenin expression.

### The *Myog* promoter is a specific target of ΔN-JMJD2A

**Figure 4:**
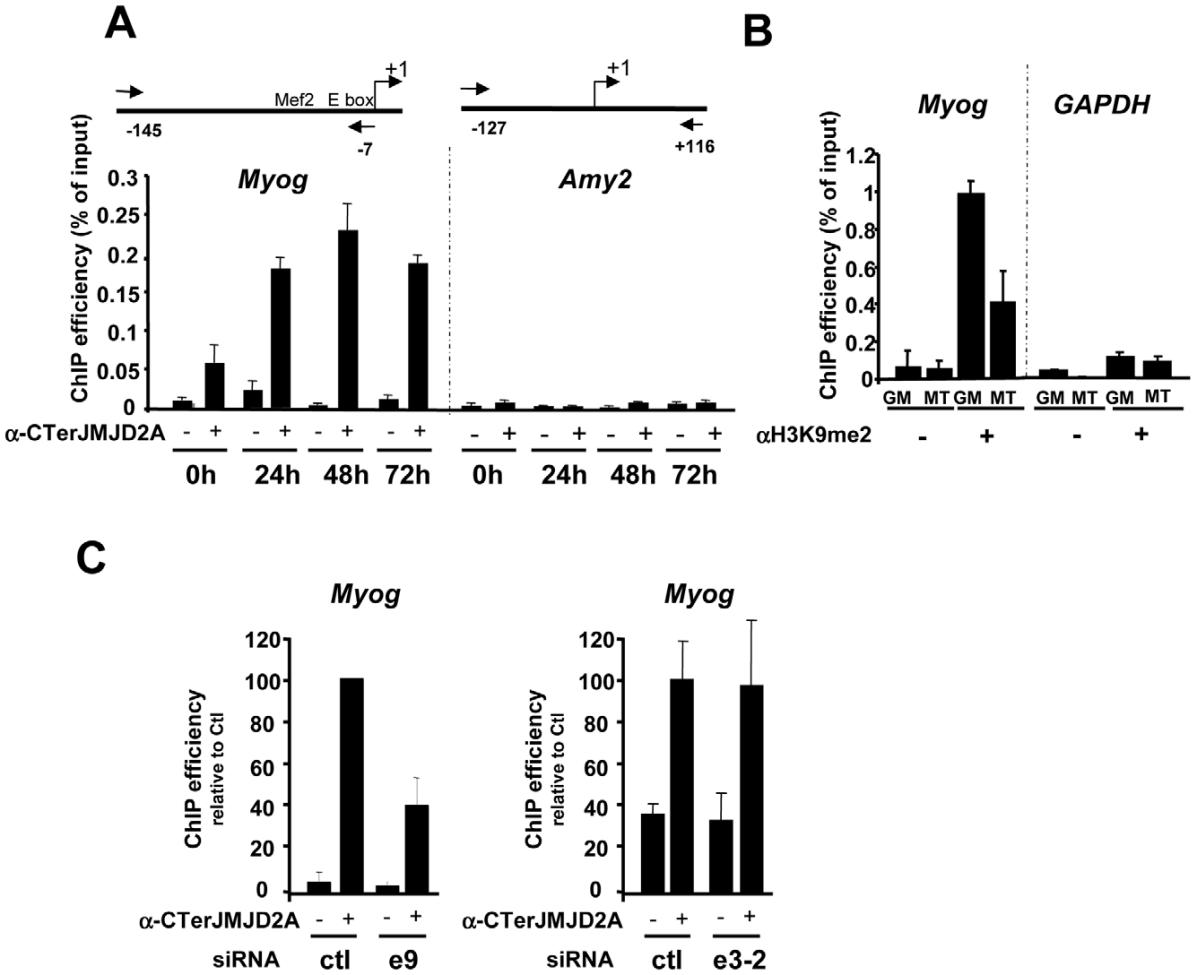
ΔN-JMJD2A is recruited to the myogenin promoter. (A) ChIP assays were performed on C2C12 cells before (0 h) or after 24, 48 or 72 hours of differentiation by using an anti-JMJD2A antibody (+) (α-Cter antibody), or no antibody (−). *Myog* and *Amy2* promoters were quantified by qPCR. The data shown indicate ChIP efficiency as a proportion of the input. Position of the primers used are indicated (B) ChIP analysis of the *Myog* and *Gapdh* promoters immunoprecipitated by using anti-H3K9me2 antibody (αH3K9me2, +), or no antibody (−) on chromatin prepared from proliferating (GM) or differentiating (72 hours) C2C12 cells (MT), as quantified by qPCR. Results represent ChIP efficiency according to input. (C) C2C12 myoblasts were electroporated with an siRNA targeting both isoforms of JMJD2A (e9), a siRNA targeting the full-length JMJD2A only (e3-2) or a control siRNA (ctl). Twenty-four hours later, cells were placed in differentiation medium for one day. ChIP experiments were performed as in (A). The data represent ChIP efficiency (%) relative to the amount of *Myog* promoter immunoprecipitated by the anti-JMJD2A antibody.from control siRNA electroporated cells.

Since ΔN-JMJD2A functions upstream of myogenin expression in the control of C2C12 myoblast cell differentiation, and *Myog* is a master gene regulating muscle differentiation, we reasoned that the *Myog* promoter might be an important target of ΔN-JMJD2A. To investigate this possibility, we performed chromatin immunoprecipitation (ChIP) on chromatin from both proliferating and differentiating C2C12 cells by using an antibody against the C-terminus of JMJD2A, which recognizes both isoforms, and the immunoprecipitated *Myog* promoter was quantified by qPCR. (The amylase promoter was used as a negative control since the amylase gene is not expressed in muscle cells.) We found the *Myog* promoter specifically in JMJD2A-ChIPed chromatin from differentiating C2C12 cells within 24 hours of differentiation (Figure 4A), a time at which most cells already expressed myogenin (as observed by immunofluorescence microscopy; data not shown). By contrast, in proliferating cells, in which myogenin is not expressed, the signal from the *Myog* promoter was just above or below the threshold of detection in some experiments). No signal from the amylase promoter was observed (Figure 4A). The amount of dimethylated H3K9 (H3K9me2) associated with the *Myog* promoter in proliferating cells was higher than in differentiating cells (Figure 4B); this decrease in differentiating cells is not due to changes in nucleosome occupancy (Figure S3). No change in the amount of H3K9me2 was observed at the *GAPDH* promoter, used as a control. Thus, binding of JMJD2A (whether FL-JMJD2A, ΔN- JMJD2A or both) to the *Myog* promoter correlates with demethylation of H3K9me2 and transcriptional activation of the gene.

**Figure 5:**
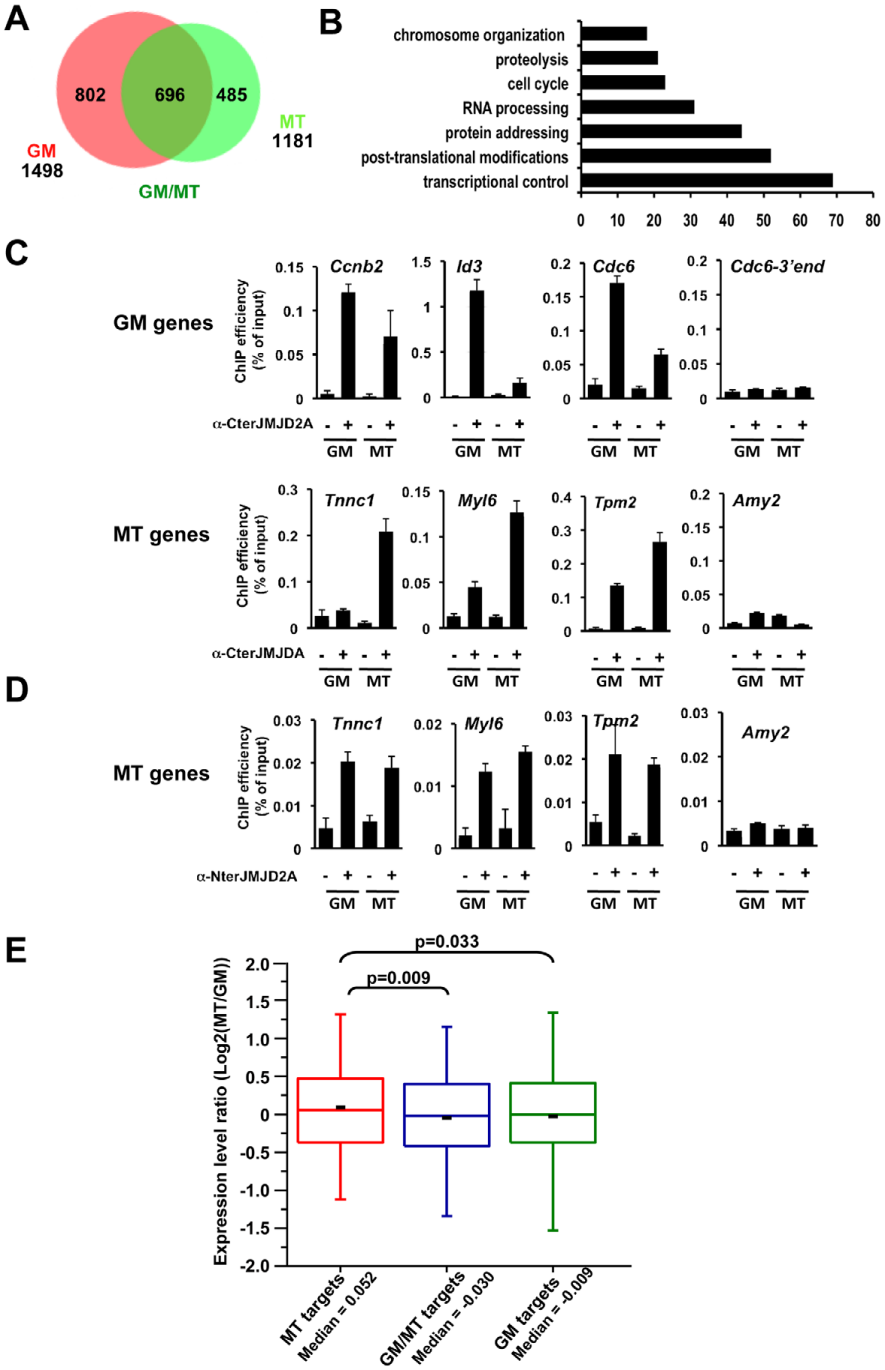
Genome-wide analysis of JMJD2A-associated promoters. (A) ChIP-on-chip analysis (as described in Text S1) of chromatin from proliferating C2C12 cells (GM), or after 72 hours of differentiation (MT). ChIP assays were performed as in Figure 4 by using the anti-JMJD2A antibody recognizing both isoforms (α-Cter antibody). Numbers of JMJD2A targets in proliferatng (GM) or differentiated (MT) cells are represented on the Venn diagram. (B) Gene ontology analysis of genes bound specifically by JMJD2A in differentiating cells. (C) Validation by qPCR of selected genes identified by ChIP-on-chip analysis. Results represent ChIP efficiency according to input. (D) MT-specific genes analysed in (C) were subjected to ChIP analysis with chromatin prepared as in (A) by using the anti-JMJD2A antibody recognising FL-JMJD2A only (α-Nter antibody). Results represent ChIP efficiency according to input. (E) Distribution of expression data from Blais *et al.,* 2005 according to the binding of JMJD2A. Box plots illustrate the genes bound specifically in differentiating cells (MT), in proliferative cells (GM) or in both conditions (GM/MT). The ratio of mRNA expression between myotubes and myoblasts are plotted. The bottom and top of the box correspond to the 25% and 75% cut off respectively, the band is the median, the dash represents the mean value of the group and whiskers correspond to the 5^th^ and the 95^th^ percentiles. Statistical analyses of observed differences are indicated (p values, student T test).

To test whether the *Myog* promoter is a specific target of ΔN-JMJD2A, we performed ChIP with the antibody against the C-terminus of JMJD2A following transfection of siRNAs that interefere specifically either only with FL-JMJD2A or with both isoforms. We found that JMJD2A binding to the *Myog* promoter was significantly reduced in cells transfected with an siRNA (e9) targeting both isoforms, but was not affected by an siRNA targeting only FL-JMJD2A (e3-2; Figure 4C). By contrast, this e3-2 siRNA decreased the signal from the *Tpm2* promoter (Figure S4), one of the genes that was most enriched in ChIPs performed by using an antibody specific for FL-JMJD2A (see Figure 5D). These data indicate that the *Myog* promoter is a direct target of the ΔN-JMJD2A isoform and probably accounts for its role in C2C12 differentiation of C2C12 myoblasts.

### Genome-wide analysis of JMJD2A targets

We wanted to identify other genes that may be targets of ΔN-JMJD2A, however, without an antibody specific for the ΔN-JMJD2A isoform, we could not ChIP this isoform independently of FL-JMJD2A. Since ΔN-JMJD2A increases during differentiation, whereas FL-JMJD2A does not (Figure 1B), we decided to search by ChIP for genes that were bound by JMJD2A in differentiated C2C12 cells but not in undifferentiated cells. Immunoprecipitated DNA fragments were amplified and hybridized to a microarray covering about 20,000 mouse gene promoters (ChIP-on-chip methodology). Hybridization and bioinformatics analysis allowing identification of bound regions were done by Nimblegen using the Roche Nimblescan software. We scored promoters as bound by JMJD2A if a binding peak (FDR p<0.01) was identified between −2000 and +500 bp from the transcription start site in at least three of four independent experiments. We found JMJD2A bound to 1498 genes in proliferating cells and 1181 in differentiated cells (see Table S2 for the complete list of JMJD2A-bound promoters). Of note, we did not identify the Myog promoter in this analysis although we know from the experiments described above (Figure 4A) that it is bound by JMJD2A, suggesting that we may have missed some JMJD2A target genes in our analysis. When we used a slightly less stringent analysis, we did find the Myog promoter among the promoters bound by JMJD2A specifically in myotubes (p<0.05; data not shown). Subsequent analysis by ChiP and qPCR on selected genes, however, indicated that, although identifying *bona fide* JMJD2A target genes, the lower stringency analysis did not reflect the actual variation in JMJD2A occupancy in myotubes versus myoblasts. We thus concentrated on the lists of genes shown in Table S2.

We found 485 genes bound by JMJD2A in differentiated cells but not in proliferating cells (Figure 5A and Table S2) that are candidates for specific targets of ΔN-JMJD2A. From this list, we selected eight genes for further analysis by ChIP and qPCR (*Tnnc1*, *Hfe2*, *Zfp238*, *Ttn*, *Tpm2*, *Fyn*, *cdkn1a* and *myl6*; Figure 5C and Figure S5). All these genes either bound by JMJD2A specifically in myotubes or were more enriched in mature myotubes than in growing myoblasts. We also analyzed by ChiP and qPCR three genes identified in the ChIP-on-chip approach bound by JMJD2A specifically in proliferating cells (*cdc6*, *Id3* and *cnnb2*) and found they were indeed preferentially bound by JMJD2A in proliferating cells. We conclude that our ChIP-on-chip experiments identified *bona fide* targets of JMJD2A and that the genes included in the Venn diagram represent JMJD2A target genes preferentially enriched in either growing myoblasts (GM), differentiated myotubes (MT) or both (Figure 5A). Gene ontology analysis of the 485 genes preferentially bound in myotubes indicated that they are mainly involved in gene regulation and signaling (Figure 5B), consistent with a role of JMJD2A in controlling cell fate. To confirm that these genes are bound by ΔN-JMJD2A, we performed ChIP experiments on chromatin from proliferating or differentiated cells by using an antibody that recognizes only the full-length isoform (α-Nter antibody; Figure S6). Specific enrichment by using this antibody was too low to perform accurate ChIP-on-chips experiments, and we analyzed by qPCR the binding of FL-JMJD2A to promoters of the previously validated genes. FL-JMJD2A bound specifically to all the genes we tested except *Hfe2*, but not to the amylase promoter used as a negative control (Figure 5D and Figure S5). There was no significant difference in the signals from myoblasts and myotubes. These data suggest that recruitment of ΔN-JMJD2A accounts, at least in part, for the increase in total JMJD2A binding detected with the antibody that recognizes both isoforms. We verified this conclusion for the *Tnnc1* promoter by knocking down JMJD2A with an siRNA against both isoforms or an siRNA against only FL-JMJD2A: the siRNA against both isoforms decreased JMJD2A binding to the promoter, whereas the siRNA against FL-JMJD2A only did not (Figure S7).

Previous transcriptional profiling studies of differentiating C2C12 cells found that expression of the eight genes we validated by qPCR is induced during differentiation [24], [25].

To evaluate the impact of ΔN-JMJD2A binding on global gene expression, we compared our genome-wide JMJD2A-binding data with mRNA expression profiles of differentiating C2C12 cells previously reported by Blais and collaborators (Figure 5E). The fold change in transcripts levels in myotubes *versus* myoblasts was assigned to the genes specifically bound by JMJD2A. We found that the genes that were specifically bound by JMJD2A in myotubes (a group enriched in genes targeted by ΔN-JMJD2A) had increased mRNA levels during differentiation, compared to the genes bound by JMJD2A in myoblasts only (GM) or in both situations (GM/MT). These data show that a subset of JMJD2A-binding genes are targeted by ΔN-JMJD2A in myotubes and this correlates with their transcriptional activation, as previously observed for *Myog* (see Figure 1 and Figure 4A). This suggests that ΔN-JMJD2A functions as a transcriptional co-activator at certain promoters.

### ΔN-JMJD2A allows the removal of repressive chromatin marks

**Figure 6:**
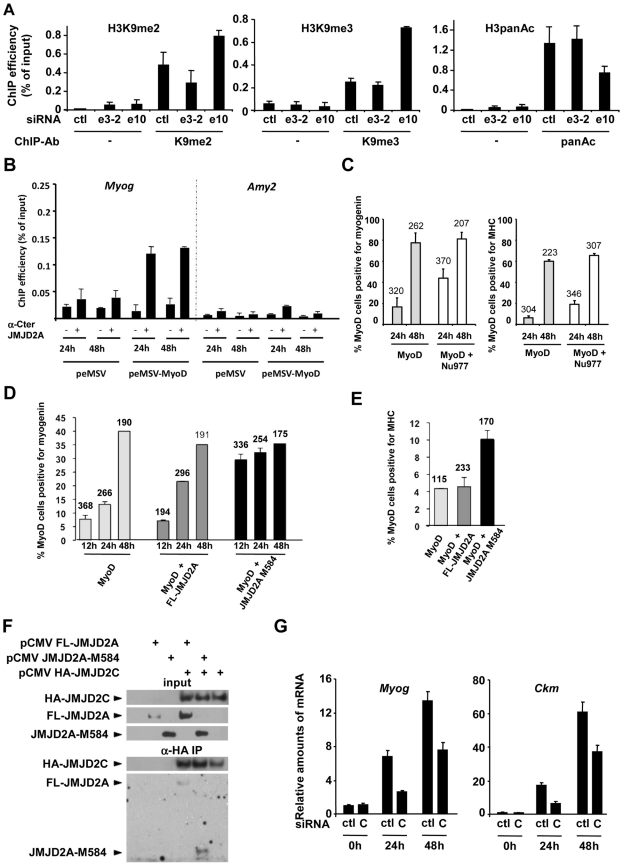
ΔN-JMJD2A enables the removal of a repressive chromatin state. (A) C2C12 myoblasts were transfected with an siRNA targeting both isoforms of JMJD2A (e10), a siRNA targeting the full-length only (e3-2) or a control siRNA (ctl). Twenty-four hours later, cells were placed in differentiation medium for one day. Chromatin was prepared and subjected to ChIP using an anti-H3K9me2 antibody (left), anti-H3K9me3 antibodies (middle) or an anti-H3panAc antibody (right). *Myog* promoter DNA was quantified by qPCR. Results represent ChIP efficiency according to input. (B) C3H10T1/2 cells were transfected with 1 µg of pEMSV-MyoD or empty pEMSV as a control. Twenty-four hours later, they were placed in differentiation medium for 48 hours. *Myog* and *Amy2* promoters in the ChIPs with anti-JMJD2A antibody (α-Cter antibody, +) or no antibody (−) were quantified by qPCR. Results represent ChIP efficiency according to input. (C) NIH3T3 cells were transfected with plasmids encoding MyoD (pEMSV-MyoD) and JMJD2A-Nu977 (pCDNA3-JMJD2A-Nu977). After 24 and 48 hours of differentiation, expression of MyoD, myogenin and myosin heavy chain (MHC) was analysed by immunofluorescence microscopy. The data represent the percentage of MyoD-expressing cells that were also positive for myogenin (left) or MHC (right). The number of cells counted in each condition is indicated above the histograms. (D) NIH3T3 cells were transfected with plasmids encoding MyoD (pEMSV-MyoD, 0.5 µg), and 0.3 µg of FL-JMJD2A-HA (full-length), JMJD2A-M584-HA (N-terminal deletion mutant) or empty vector, as indicated, and then incubated in differentiation medium for 12, 24 or 48 hours. Expression of MyoD, HA and myogenin was analysed by immunofluorescence microscopy. Quantification represents the percentage of MyoD-expressing cells that were also positive for myogenin. The number of cells counted for each condition is indicated above the histograms. (E) As in (D), except that cells were transferred to differentiation medium for 24 hours before being stained for MyoD, HA and MHC. (F) U2OS cells were transfected with the indicated expression vectors. Fourty-eight hours after transfection, total protein extracts were prepared and immunoprecipitated with an anti-HA antibody. Inputs and immunoprecipitates were western blotted with antibodies against HA or JMJD2A. (G) C2C12 cells were transfected with an siRNA targeted against JMJD2C (C) or a control siRNA (ctl), and kept in growth medium (0 h) or transferred to differentiation medium for 24 or 48 hours. *Myog* and *Ckm* mRNA expression were quantified by RT-qPCR, standardized according to *gapdh* mRNA levels, and calculated relative to 1 in proliferating cells transfected by the control siRNA.

To understand the mechanism by which ΔN-JMJD2A promotes transcription, we first focused on the *Myog* promoter, which we identified as a direct target of ΔN-JMJD2A (Figure 4). We transfected differentiating C2C12 cells with siRNAs against both JMJD2A isoforms or against FL-JMJD2A only and analyzed the *Myog* promoter for various histone modifications: depletion of JMJD2A resulted in increased H3K9me2 and H3K9me3 and decreased H3 pan-acetylation (Figure 6A). Similar data were obtained for H3K9me2 and H3K9me3 at the *Tnnc1* promoter (Figure S7), which is also bound specifically by ΔN-JMJD2A (Figure S7). This indicates that ΔN-JMJD2A regulates histone H3K9 methylation and histone H3 acetylation levels at some muscle-specific promoters (such as the *Myog* promoter) during muscle differentiation. The differentiation-linked increase in ΔN-JMJD2A levels and the subsequent increase in promoter-bound protein are probably responsible, at least in part, for changing the chromatin at muscle-specific genes from a repressive to an activating state during terminal differentiation, allowing transcriptional activation of the genes.

To investigate directly whether ΔN-JMJD2A counteracts silencing of muscle-specific promoters, we used a myogenic conversion assay in which non-muscle cells are converted into muscle cells by expression of the myogenic transcription factor MyoD. During this process, the chromatin containing muscle genes shifts from a silent to an active state. We transfected mouse C3H10T1/2 fibroblasts with MyoD to induce muscle conversion and used the α-Cter antibody against JMJD2A in ChIP assays to test whether JMJD2A was recruited to the *Myog* promoter (Figure 6B). We found JMJD2A bound to the *Myog* promoter one day after passage into differentiation medium, and remained at that same level one day later. (No binding was detected at the amylase promoter, used as a negative control.) Thus, JMJD2A is recruited to the *Myog* promoter during the process of myogenic conversion. We next investigated whether ΔN-JMJD2A would potentiate myogenic conversion. NIH3T3 cells were transfected with an expression vector encoding MyoD either alone or in combination with an expression vector producing the endogenous small mRNA beginning at nucleotide +977 (JMJD2A-Nu977), which can encode ΔN-JMJD2A (see Figure 2C). Twenty-four hours after transfection, cells were shifted to differentiation medium for 24 h or 48 h and analyzed by immunofluorescence microscopy for expression of MyoD, myogenin and myosin heavy chain (MHC). As estimated by the percentage of MyoD-positive cells that co-expressed myogenin or MHC, most cells converted to muscle 48 h after passage into differentiation medium, irrespective of the presence of JMJD2A-Nu977 mRNA expression vector (Figure 6C). Myogenic conversion was significantly enhanced, however, by JMJD2A-Nu977 mRNA overexpression at the 24 h point, with a 2.5-fold increase in cells expressing myogenin (p<0.02), and a 3-fold increase in cells expressing the late differentiation marker myosin heavy chain (p<0.01) (Figure 6C). To see if this pro-myogenic activity of JMJD2A-Nu977 mRNA was isoform-specific, we performed similar assays by co-expressing HA-tagged FL-JMJD2A or JMJD2A-M584 (which encodes a protein very similar in size to ΔN-JMJD2A; see Figure 2) with MyoD. In addition to MyoD, we stained cells either for myogenin or for HA-JMJD2A and MHC (see Figure S8 for typical images). We observed that 70–80% of cells stained for both MyoD and the HA epitope (data not shown). Co-staining for MyoD and myogenin was performed on cells that had been differentiating for 12, 24 and 48 hours. Twelve hours after passage into differentiation medium, the percentage of cells that were positive for myogenin was much greater in the cells expressing JMJD2A-M584 than in the cells transfected with MyoD alone, whereas there was little difference between the cells expressing FL-JMJD2A and the cells transfected with MyoD alone (Figure 6D). Similarly, in cells expressing MyoD alone 24 h after the shift into differentiation medium about 5% of MyoD-positive cells expressed MHC. This percentage was unchanged in cells co-expressing HA-FL-JMJD2A, but enhanced more than two-fold in cells co-expressing JMJD2A-M584 (Figure 6E). We conclude that ΔN-JMJD2A is a positive regulator of MyoD-induced differentiation and functions by favoring the transition of muscle-specific promoters from a repressed to an active state.

JMJD2A is reported to interact with another histone demethylase JMJD2C [26]. To test whether ΔN-JMJD2A retains this binding ability, we transfected U2-OS osteosarcoma cells with expression vectors for HA-JMJD2C and either FL-JMJD2A or JMJD2A-M584. We immunoprecipitated JMJD2C and found both FL-JMJD2A and JMJD2A-M584 co-immunoprecipitated (Figure 6F), indicating that JMJD2A-M584 retains the ability to interact with JMJD2C. This suggests that ΔN-JMJD2A favors H3K9 demethylation by physically interacting with the catalytically active JMJD2C-demethylase. Consistent with this, we found that depletion of JMJD2C by using specific siRNAs (see Figure S9 for the characterization of JMJD2C siRNA) recapitulates the effects of ΔN -JMJD2A inactivation on induction of the *Myog* and *Ckm* genes (Figure 6G).

## Discussion

We report here the existence of an isoform of JMJD2A (ΔN-JMJD2A) that is expressed during skeletal muscle differentiation and is required for this process. Moreover, we identify the *Myog* promoter as a specific target of this isoform and demonstrate that it mediates demethylation of histone H3 at residue K9 at this promoter. Our findings suggest a model in which expression of ΔN-JMJD2A and its consequent binding to the *Myog* promoter mediates derepression of the *Myog* gene, a key event during the terminal differentiation of skeletal muscle, by promoting demethylation of the promoter.

### A small isoform of JMJD2A is upregulated during muscle differentiation

This new isoform of the JMJD2A demethylase we identify here lacks the N-terminal part of the full-length protein, which contains the demethylase domain. We show that in C2C12 cells, it is produced from a mRNA that initiates at exon 8. We found no description in several databases (Genbank, UCSC) of alternative splicing of the JMJD2A gene that might give rise to the ΔN-JMJDA variant, suggesting that an internal promoter might give rise to the shorter mRNA, which may be regulated in a differentiation-dependent manner. Moreover, initiation through an internal promoter would be consistent with the description of a short RNA in the GENBANK database. Note however that the 5′ end we identified by 5′RACE (nucleotide 977 of the full-length sequence) is upstream of that described in the GENBANK database for the short RNA. Our data from using exon-specific siRNA to knock-down JMJD2A are consistent with the interpretation that nucleotide 977 constitutes the transcription start site: only siRNAs targeting exons downstream of exon 9 decreased both JMJD2A-FL and ΔN-JMJD2A expression and inhibited differentiation.

Endogenous ΔN-JMJD2A protein is certainly very similar (although perhaps not strictly identical) to JMJD2A initiating at Met584 (JMJD2A-M584), since the two proteins migrate at very similar molecular weights on SDS-PAGE and behave similarly in enhancing MyoD-induced muscle differentiation of non-muscle cells. An important question is how ΔN-JMJD2A protein is produced from the Nu977 mRNA. When overexpressed in C2C12 cells, JMJD2A-Nu977 mRNA (but not the full-length construct) is translated from three distinct initiator codons encoding methionine residues 317, 427 and 584. This probably reflects the so-called ‘leaky scanning of ribosomes’ mechanism described recently by Calkhoven and collaborators [27]. This mechanism is controlled by specific signal transduction pathways and results in an accurate ratio of CEBP isoforms critical for cell fate control [27]. Although the intermediate forms of JMJD2A are observed only upon overexpression of the JMJD2A-Nu977 mRNA, a ‘leaky scanning of ribosomes’ mechanism might participate in the production of the ΔN-JMJD2A isoform. Alternate but not mutually exclusive explanations could be that ΔN-JMJD2A is produced through an ‘IRES’-type mechanism requiring a special structure adopted by the 5′ end of the nu977 mRNA, or by limited proteolysis of a larger protein. In agreement with this latter hypothesis, there is no initiator codon that would give rise to a protein with precisely the molecular weight of ΔN-JMJD2A. Whatever the mechanism, it is regulated in a differentiation-dependent manner, since the expression of ΔN-JMJD2A increases with differentiation although expression of JMJD2A-Nu977 mRNA does not, and overexpression of JMJD2A-Nu977 produces much more ΔN-JMJD2A in differentiating cells than in proliferating cells.

### ΔN-JMJD2A is essential for differentiation and acts as an activator

We show that the *Myog* promoter is a direct target of ΔN-JMJD2A. Recruitment of ΔN-JMJD2A correlates with *Myog* transcriptional activation at the onset of differentiation, suggesting that its acts as a transcriptional activator. Our evidence that inactivation of ΔN-JMJD2A by specific siRNAs affects the induction of *Myog* transcription during muscle differentiation supports this conclusion, as does the significant association between myotube-specific targets, identified by our ChIP-on-chip approach, and genes induced during differentiation [24], [25]. FL-JMJD2A has been described previously as a co-repressor of both ASCL2- [12] and E2F-targeted genes [13], in association with N-CoR and Rb respectively, suggesting that ΔN-JMJD2A may have a dominant negative effect on FL-JMJD2A. This is unlikely because siRNA knockdown of FL-JMJD2A does not noticeably affect the differentiation process or myogenin expression. These observations suggest that ΔN-JMJD2A might function independently of FL-JMJD2A by recruiting specific partners. One might imagine, for example, that FL-JMJD2A folds in such a way that binding sites for partner proteins in the C-terminal part of the protein are masked by the N-terminal sequences, a masking that would not occur for the ΔN-JMJD2A isoform.

### Role of ΔN-JMJD2A on the *Myog* promoter

The requirement of ΔN-JMJD2A for differentiation may be explained by its role on myogenin expression. Myogenin is a key effector of muscle differentiation and is required for late differentiation events [16]. We show that ΔN-JMJD2A directly regulates the *Myog* gene, being recruited to its promoter concomitantly with transcriptional activation in both C2C12 cells and MyoD-converted fibroblasts. Although this latter finding suggests that MyoD might target ΔN-JMJD2A to the *Myog* promoter, we found no interaction between ΔN-JMJD2A and MyoD. Moreover, the fact that genes identified in our ChIP-on-chip analysis are not enriched in MyoD-targeted genes argues against a role for MyoD in this process [16]. ΔN-JMJD2A contains a Tudor domain in its C-terminal part [28] that might recruit the protein to its target genes by interacting with methylated H3K4, a modification enriched in the promoter region of active genes, including *Myog* [20]. H3K4 methylation, being generally associated with RNA Polymerase II-dependent gene activation [29], other mechanisms must be involved in recruiting ΔN-JMJD2A specifically to its target promoters. The mechanisms controlling recruitment of ΔN-JMJD2A to the *Myog* promoter are likely critical for myoblasts to undergo terminal differentiation and deserve further analysis to understand how JMJD2A discriminates between its target genes. As we found that MEF2A or MEF2C expression is required for ΔN-JMJD2A recruitment to the *Myog* promoter, MEF2 may be part of this process.

Although the molecular mechanisms by which ΔN-JMJD2A activates gene expression are still unclear, we have shown that its recruitment to the *Myog* promoter is required for demethylating H3K9, an event previously shown to correlate with *Myog* activation. H3K9 methylation at the *Myog* promoter is catalysed by Suv39h1/KMT1A associated with MyoD [23]. This process is positively regulated by the protein kinase p38-γ that favors the association of KMT1A with MyoD by phosphorylating MyoD at Ser199/200. As a result, levels of methylated H3K9 at the *Myog* promoter increase and both muscle differentiation and MyoD-induced myogenic conversion of fibroblasts are impaired [22]. Thus, methylation of H3K9 is a central to *Myog* gene repression in myoblasts. It impedes premature entry into the differentiation process and allows expansion of undifferentiated cells, a process that has to be tightly controlled during both muscle development and regeneration. We show that demethylation of H3K9 at the myoblast-to-myotube transition depends on ΔN-JMJD2A since neither accurate *Myog* induction nor H3K9 demethylation was observed in ΔN-JMJD2A depleted cells.

Because ΔN-JMJD2A lacks a demethylase domain, it must recruit another H3K9-specific demethylase. Our findings, together with previously published data, indicate that this demethylase might be JMJD2C: ΔN-JMJD2A physically interacts with JMJD2C and depletion of JMJD2C recapitulates the effects of ΔN-JMJD2A depletion. H3K9me3 is the preferred substrate of JMJD2C [3], [7], although we show here that ΔN-JMJD2A is important for demethylation of both H3K9me3 and H3K9me2. When recruited to the *Myog* promoter, JMJD2C might also demethylate H3K9me2, as it does when it is overexpressed [3], [26]. Alternatively, another K9me2-specific demethylase, such as LSD1, might be involved. LSD1 was recently found to target the *Myog* promoter [30].

Another important epigenetic regulator of muscle differentiation, MITR/HDRP also lacks a catalytic domain; it comprises the non-catalytic N-terminal region of HDAC9 and is generated by alternative splicing [31]. MITR is highly expressed in skeletal muscle [32]; it is involved in repressing MEF2-dependent genes through histone deacetylation of their promoters by recruiting other deacetylases [33]. This example illustrates the importance of variants of chromatin modifying enzymes and, in particular, of isoforms that are deficient in enzymatic activity. Strikingly, isoforms of many other epigenetic regulators have been identified or are suspected to exist. Of note, the *LSD1* gene was recently shown to produce four alternatively spliced isoforms, some of which are specifically expressed in neurons and play roles in neurite morphogenesis [34]. A study by Lois and collaborators [35] indicates that 49% of chromatin modifiers have alternative spliced variants and that in more than 59% of cases, splicing affects either the catalytic domain or the interaction domain. The importance of isoforms of chromatin modifying enzymes may be explained by the existence of multimolecular complexes containing many chromatin modifying activities: by incorporating catalytically active or defective isoforms of a given enzyme, these complexes may specify distinct histone codes at their target sites, leading to different transcriptional outcomes.

## Materials and Methods

### Cell culture

The C2C12 skeletal myoblast cell line was cultured under standard conditions in DMEM-F12 supplemented with 15% FCS (Gibco). C3H10T1/2 were maintained in MEM supplemented with 15% FCS. NIH3T3 and U2-OS cells were grown in DMEM supplemented with 10% FCS (Gibco). To induce differentiation, cells were placed in differentiation medium (DMEM supplemented with 1.5% horse serum (Invitrogen)). Human skeletal muscle satellite cells were a gift from Gilles Carnac (Montpellier, France) and were cultured as previously described [36].

### JMJD2A constructs, transfection, and electroporation

Details of the construction of FL-JMJD2A and N-terminal deletion mutants will be provided upon request. Cells were grown in six-wells plates and transfected by using Reagent+/Lipofectamine (Invitrogen) according to the manufacturer's instructions. One microgram of mixed plasmids was used for each transfection. In control experiments, 60–70% of NIH3T3 cells and 40–50% of C3H10T1/2 cells transfected with pEGFPN1 (Clontech) expressed GFP. For siRNA transfection, 4×10^6^ C2C12 myoblasts were electroporated with double-stranded siRNA (purchased from Eurogentec or Dharmacon and described in Text S1) at a final concentration of 8 µM by using an eletroporation device (Amaxa AG, Cologne, Germany) according to the manufacturer's instructions. Differentiated cells were transfected by using oligofectamine (Invitrogen).

### Western blotting

Cells were lysed for western blotting by incubating them with Laemmli sample buffer (63 mM Tris pH 6.8, 8% glycerol, 2.5% SDS, bromophenol blue, 5% ß-mercaptoethanol) for 5 min at 85°C to denature proteins. Western blots were performed using standard procedures and antibodies were used at the following concentrations: rabbit polyclonal anti-Cter JMJD2A 0.4 µg/mL (A300861A, BethylLab), rabbit polyclonal anti-Nter JMJD2A 0.1 µg/mL (HPA007610, Sigma), mouse monoclonal anti-myogenin 0.4 µg/mL (F5D, Santa-Cruz), mouse monoclonal anti-α-tubulin 0.1 µg/mL (T6199, Sigma), mouse monoclonal anti-MHC 0.1 µg/mL (05–716, Upstate), mouse monoclonal anti-HA diluted 1/1000 (HA11 Babco, Covance). Peroxidase-conjugated secondary antibodies were purchased from Amersham.

### Immunofluorescence microscopy

Cells were fixed by adding formaldehyde to a final concentration of 3.7% in PBS and permeabilized in PBS containing 0.5% Triton X100. The fixed and permeabilized cells were incubated at 4°C overnight with primary antibodies at the following concentrations: rabbit polyclonal anti-Nter JMJD2A 0.1 µg/mL (HPA007610, Sigma), mouse monoclonal anti-myogenin 0.5 µg/mL (F5D, Santa-Cruz), rabbit polyclonal anti-MyoD 0.5 µg/mL (C-20, Santa-Cruz), mouse monoclonal anti-HA 1/1000e (HA11, Covance), mouse monoclonal anti-myosin heavy chain 0.2 µg/mL (05–716, Upstate). Fluorochrome-conjugated secondary antibodies were purchased from Invitrogen. Observations were carried out with a fluorescence microscope (DM; Leica, Wetlar, Germany) equipped with a cooled charge-coupled device camera, and images were acquired using the MetaVue imaging system (Universal Imaging Corp., West Chester, PA).

### Co-immunoprecipitation experiments

Plasmids encoding untagged forms of FL-JMJD2A or JMJD2A-M584 either alone or together with a HA-tagged form of JMJD2C (kindly provided by K. Helin, BRIC Copenhagen) were introduced into U2OS cells by using an electroporation device (Amaxa AG) according to the manufacturer's instruction at a ratio of 2 µg of plasmid for 10^6^ cells. Fourty-eight hours after transfection, cells were lysed in IP buffer (10 mM Tris pH 8, 0.4% NP40, 300 mM NaCl, 10% glycerol, 1 mM DTT, anti-protease (Roche), anti-phosphatase (Sigma) inhibitors). Lysates were diluted using one volume of dilution buffer (10 mM Tris pH 8, 0.4% NP40, 5 mM CaCl2, 2 U/mL RQ1 DNAse (Promega)) and then incubated with anti-HA antibodies coupled to agarose beads (Sigma) overnight at 4°C. Beads were washed four times with IP buffer, eluted with Laemmli sample buffer without ß-mercaptoethanol and analyzed by western blotting.

### Total RNA extraction and reverse transcription

Total RNA was extracted from cells by using the RNeasy mini kit (Qiagen). Five hundred nanograms of RNA were reverse-transcribed for 50 min at 42°C in a 20 µL reaction volume containing 0.5 µM dNTPs, 0.5 µg of random primers, 10 mM DTT, 1X AMV RT buffer, 40 U of RNasin and 10 U of AMV Reverse Transcriptase (Promega). Samples were incubated for 15 min at 70°C to stop the reaction. Samples were analyzed by qPCR by using the primers described in Text S1 on a CFX96 real-time system device (Biorad) using the platinium SYBR Green qPCR SuperMix (Invitrogen). For quantification of JMJD2A isoforms, RT-qPCR was performed by including a dilution curve of JMJD2A plasmid from 1 pg to 0.1 fg corresponding to 110000 to 11 JMJD2A mRNA molecules, allowing us to estimate the exact number of molecules per µg of total mRNA isolated either from myoblasts or myotubes.

### Expression array analysis

100 ng of total RNA for each condition was subjected to cleanup, reverse transcription, amplification and labelling according to the manufacturer's instructions (GeneChip whole transcript sense target labelling assay, Affymetrix). Raw data were processed using Genespring GX 10.0 analysis software (Agilent technologies Inc, Santa Clara, CA). Briefly, after normalization using the RMA algorithm, a t-test statistical analysis was carried out to select genes whose expression changed significantly compared to control (p-value < = 0.05) in three independent experiments. Genes were considered as regulated by JMJD2A when the average fold change relative to control was higher than 1.5. Clustering was performed according to gene ontology by using DAVID Bioinformatics Resources 6.7 (http://david.abcc.ncifcrf.gov) and manual curation.

### 5′RACE

5′RACE experiments were carried out on 10 µg of total mRNA extracted from differentiated cells, following the manufacturer instructions (First choice RLM-RACE kit, Ambion). Briefly, after decapping and 5′ dephosphorylation, an adaptor was ligated at the 5′ end of the mRNA and reverse transcription was performed using primers that hybridised with sequences in exon 11 of JMJD2A (5′-CAGACGCAGGATTCACAGAA-3′). 5′ends were then amplified by two rounds of PCR with primers that hybridised with sequences in the adaptor and in exon 9 of JMJD2A (round 1: 5′-AGAGCTCGCTCTGACTGACC-3′, round 2: 5′-TGGGCAGAGTGTGGTCAATA-3′). PCR products were cloned by using the pGEM-T Easy Vector kit (Promega) and sequenced.

### Chromatin immunoprecipitation

Chromatin immunoprecipitation assays (ChIP) were performed as previously described [37]. For JMJD2A ChIP, 100 µg and 300 µg of chromatin were immunoprecipitated with antibodies directed against the C-terminal region (A300861A, BethylLab, 1 µg/mL) or N-terminal region (HPA007610, Sigma, 1 µg/mL) of JMJD2A respectively. Ten micrograms and 5 µg of chromatin were used in ChIP experiments with antibodies against H3K9me2/H3K9me3 (Ab1220-100 Abcam 1 µg/mL, 07–462 Upstate 1 µg/mL) and H3-pan-acetyl (6599 Upstate 1 µg/mL), respectively. Samples were analyzed by qPCR by using the oligonucleotides described in Text S1.

### ChIP-on-chip analysis

ChIP-on-chip analyses were performed by NimbleGen on a mouse promoter array (MM8_RefSeq_promoter and MM8_Deluxe_Promoter_HX1; NimbleGen, Roche). The description of this analysis is given in Text S1. Bound genes were clustered according to gene ontology by using DAVID Bioinformatics Resources 6.7 (http://david.abcc.ncifcrf.gov) and manual curation.

## Supporting Information

Figure S1 Sequences alignment. Sequences of the full-length mouse JMJD2A mRNA (NM_001161823.1) and a shorter isoform (AK136085.1) were obtained from GENBANK and align to those of ΔN1-JMJD2A and ΔN2-JMJD2A 5′-ends described in our study, using the multalin software (http//multalin.toulouse. inra.fr/multalin ; Multiple sequence alignment with hierarchical clustering" F. CORPET, 1988, Nucl. Acids Res., 16 (22),10881-10890).
(0.11 MB PDF)

Figure S2 Knock-down of JMJD2A does not decrease JMJD2B or JMJD2C. C2C12 cells were transfected with the indicated siRNAs. mRNA were extracted, reverse transcribed and the amounts of JMJD2A, JMJD2B and JMJD2C cDNAs were quantified by qPCR and standardized relative to gapdh. Representative experiments are shown.
(0.02 MB PDF)

Figure S3 Activation of the *Myog* promoter is not associated with a detectable decrease in nucleosome occupancy. C2C12 cells were induced to differentiate for 72 hours and subjected to a ChIP experiment using 2 different anti-histone H3 antibodies, or no antibody as indicated. The amount of *Myog* promoter in the ChIPs was quantified by qPCR. A representative experiment is shown.
(0.03 MB PDF)

Figure S4 FL-JMJD2A is recruited to the *Tpm2* promoter. C2C12 cells were transfected with siRNAs targeting full-length JMJD2A only (sie3-2) or control siRNA (sictl). 24 h later, cells were shifted to differentiation medium for one day. Chromatin was prepared and subjected to a ChIP using anti-CTerJMJD2A antibody (ChIP-Ab, +), or no antibody (−). The *Tpm2* promoter was quantified by qPCR.
(0.03 MB PDF)

Figure S5 Validation of JMJD2A targets identified by ChIP-on-chips. *Hfe2*, *Cdkn1a*, *Zfp238*, *Ttn* and *Fyn* genes were selected from the list of target genes identified as specifically bound in myotubes by ChIP-on-chips to be further analyzed by a classical ChIP approach using antibodies directed against either the carboxy-terminal end (α-CterJMJD2A, panel A) or N-terminal part (α-NterJMJD2A, panel B) of JMJD2A. ChIP were performed with chromatin from either growing (GM) or differentiated C2C12 cells (MT).
(0.09 MB PDF)

Figure S6 Characterisation of the antibody directed against the N-terminal part of JMJD2A. Antibodies recognizing both isoforms of JMJD2A (α-Cter antibody) or the full-length protein only (α-Nter antibody) were tested by western blot on extracts from C2C12 cells over-expressing JMJD2A full length (FL-JMJD2A) or a Nterminal deletion mutant (JMJD2A-M427). The α-Nter antibody was tested by immunofluorescence in cells transfected by a siRNA targeting exon 10 of JMJD2A (sie10) or a control siRNA (ctl).
(0.31 MB PDF)

Figure S7 ΔN-JMJD2A targets the *Tnnc1* promoter. A) C2C12 cells were transfected with siRNA targeting either exon 9 or exon 3-2, induced to differentiate and subjected to a ChIP experiment using α-CTerJMJD2A antibody. The amounts of *Tnnc1* promoter in the ChIP were quantified by qPCR. A representative experiment is shown. The ChIP signal is decreased by the siRNA targeting both isoforms (sie9) but not by the siRNA targeting the full-length only (sie3-2), indicating that the *Tnnc1* promoter is mainly bound by ΔN-JMJD2A. B) Same as in A, except that ChIP was performed using anti H3K9me2 (left) or anti H3K9me3 (right) antibody.
(0.03 MB PDF)

Figure S8 Typical images of MyoD-converted cells. A) NIH3T3 cells were transfected with MyoD alone or together with plasmids encoding FL-JMJD2A or JMJD2A-M584. Following 24 h in differentiation medium, cells were stained for MyoD in red and for both MHC and the HA epitope in green. MHC is cytoplasmic whereas HA tagged JMJD2A proteins are nuclear, allowing to discriminate between the two signals. Arrows indicate cells expressing both MHC and MyoD, and (*) cells that express both MyoD, MHC and HA. Note that one cell is stained for both MHC and HA, but does not show detectable levels of MyoD. Bar = 10 µm. B) Cells were treated as in A except they were kept in differentiation medium for 12 h and stained for MyoD in red and myogenin in green. Arrows indicate cells that express both MyoD and myogenin. Bar = 10 µm.
(2.78 MB PDF)

Figure S9 Characterisation of the siRNA targeting JMJD2C. C2C12 cells were transfected with a siRNA targeting JMJD2C (C) or a control siRNA (ctl). mRNAs were extracted, reverse-transcribed and the amounts of *Jmjd2c* and *Jmjd2a* were quantified by qPCR and calculated relative to *gapdh*. A representative experiment is shown.
(0.03 MB PDF)

Figure S10 MEF2A-C expression is required for JMJD2A recruitment to the myogenin promoter. C2C12 cells were transfected with a siRNA targeting MEF2A and MEF2C or a control (ctl) siRNA. Cells were then induced to differentiate and subjected to a ChIP using the anti-CTerJMJD2A antibody. The amount of *Myog* (left) or *Tpm2* (right) promoters in the ChIP were quantified by qPCR. A representative experiment is shown.
(0.03 MB PDF)

Table S1 List of genes significantly affected by the JMJD2A siRNAs.
(0.03 MB XLS)

Table S2 JMJD2A ChIP-chips analysis. Here is shown the lists of genes bound by JMJD2A in proliferative conditions only (GM), in differentiating conditions only (MT) or in both conditions (commons (GM-MT)). Also shown is a Gene Ontology analysis of genes bound in differentiating conditions only (GO analysis).
(0.31 MB XLS)

Text S1 Supplementary Materials and Methods.
(0.05 MB DOC)
