# Supplementary figures and images for: A New Isoform of the Histone Demethylase JMJD2A/KDM4A Is Required for Skeletal Muscle Differentiation
Source: PLoS Genet. 2011 Jun 2;7(6):e1001390. doi: 10.1371/journal.pgen.1001390 (PMC3107188; doi:10.1371/journal.pgen.1001390)

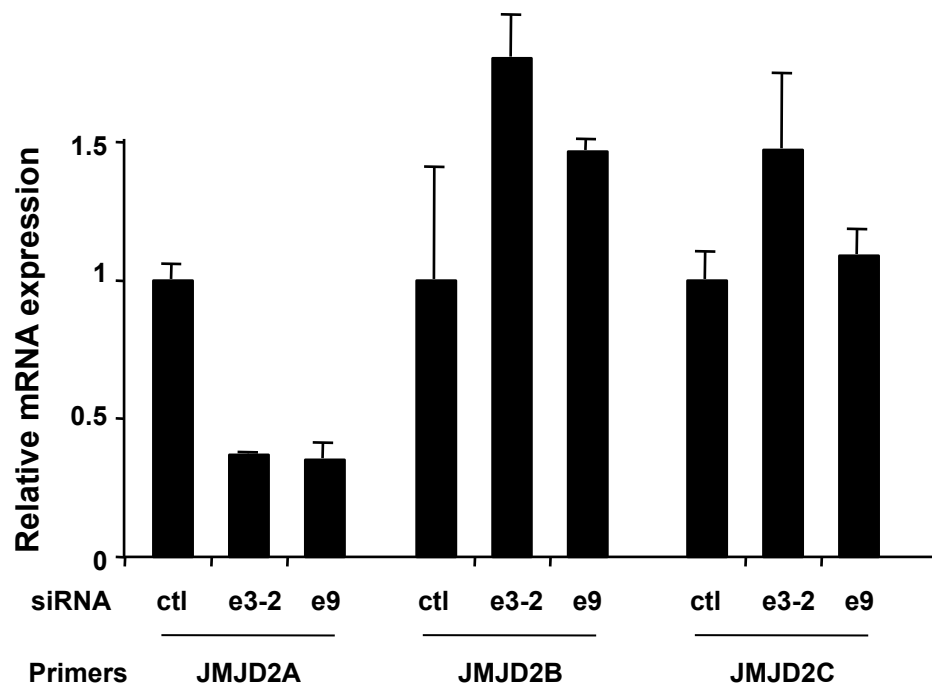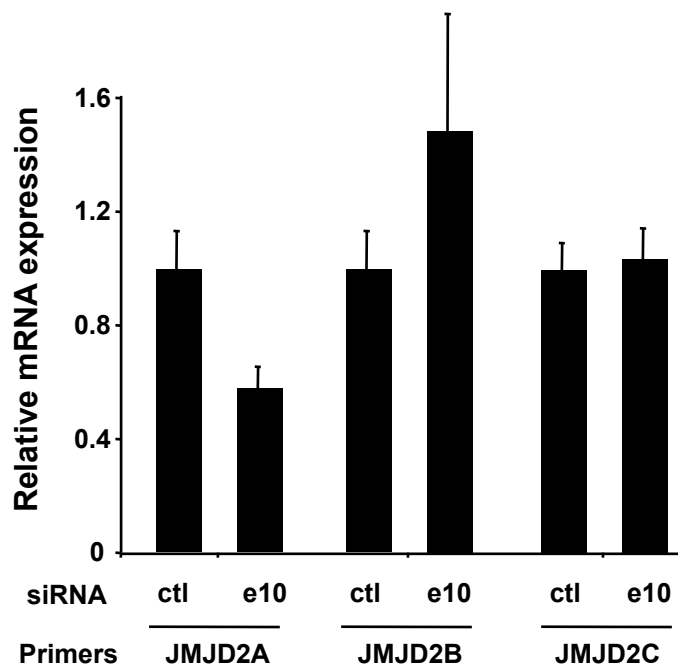

Supplement: Figure S2 — Knock-down of JMJD2A does not decrease JMJD2B or JMJD2C. C2C12 cells were transfected with the indicated siRNAs. mRNA were extracted, reverse transcribed and the amounts of JMJD2A, JMJD2B and JMJD2C cDNAs were quantified by qPCR and standardized relative to gapdh. Representative experiments are shown. (0.02 MB PDF) [file pgen.1001390.s002.pdf]

*Myog*

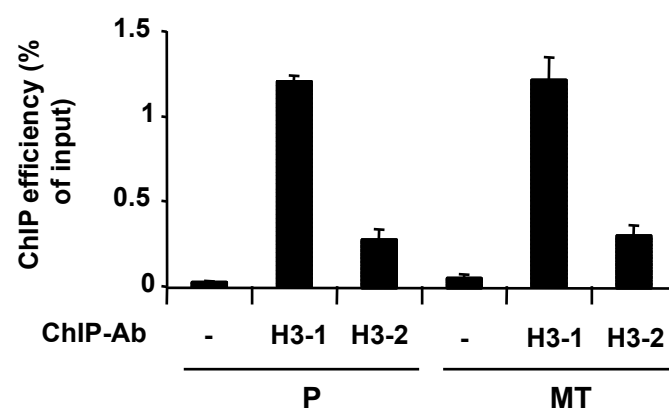

Supplement: Figure S3 — Activation of the Myog promoter is not associated with a detectable decrease in nucleosome occupancy. C2C12 cells were induced to differentiate for 72 hours and subjected to a ChIP experiment using 2 different anti-histone H3 antibodies, or no antibody as indicated. The amount of Myog promoter in the ChIPs was quantified by qPCR. A representative experiment is shown. (0.03 MB PDF) [file pgen.1001390.s003.pdf]

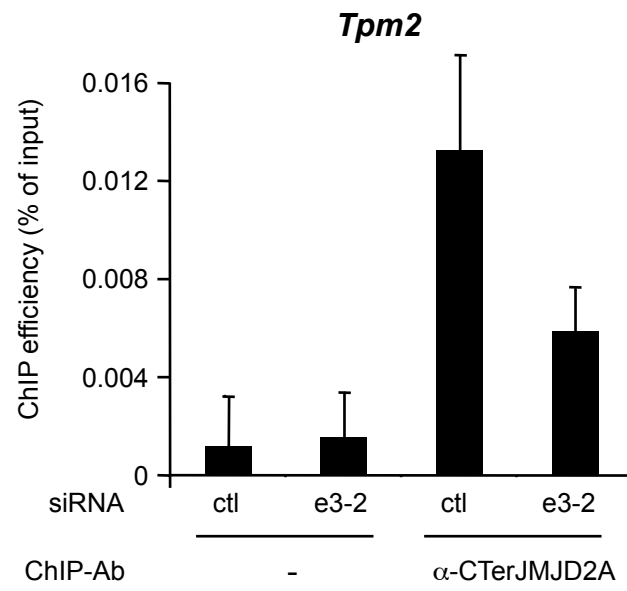

Supplement: Figure S4 — FL-JMJD2A is recruited to the Tpm2 promoter. C2C12 cells were transfected with siRNAs targeting full-length JMJD2A only (sie3-2) or control siRNA (sictl). 24 h later, cells were shifted to differentiation medium for one day. Chromatin was prepared and subjected to a ChIP using anti-CTerJMJD2A antibody (ChIP-Ab, +), or no antibody (−). The Tpm2 promoter was quantified by qPCR. (0.03 MB PDF) [file pgen.1001390.s004.pdf]

**A**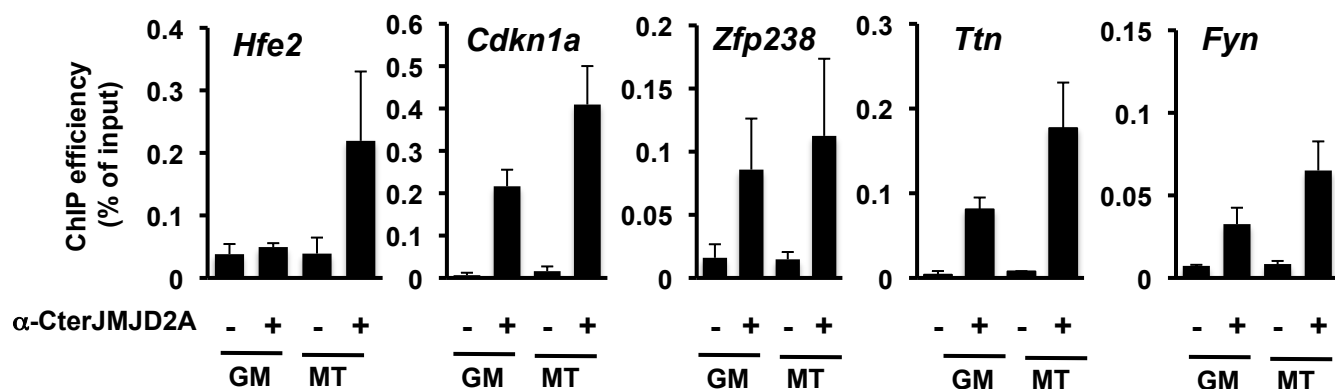**B**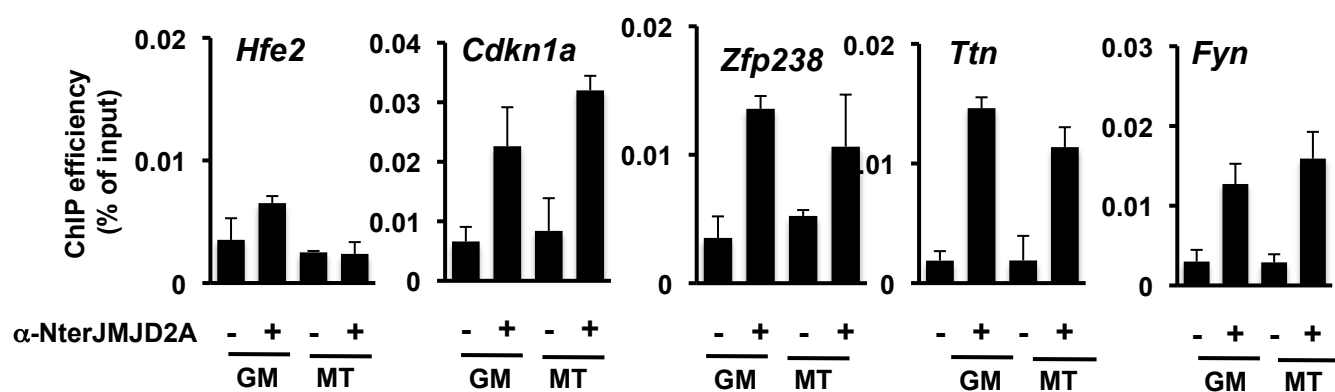

Supplement: Figure S5 — Validation of JMJD2A targets identified by ChIP-on-chips. Hfe2, Cdkn1a, Zfp238, Ttn and Fyn genes were selected from the list of target genes identified as specifically bound in myotubes by ChIP-on-chips to be further analyzed by a classical ChIP approach using antibodies directed against either the carboxy-terminal end (α-CterJMJD2A, panel A) or N-terminal part (α-NterJMJD2A, panel B) of JMJD2A. ChIP were performed with chromatin from either growing (GM) or differentiated C2C12 cells (MT). (0.09 MB PDF) [file pgen.1001390.s005.pdf]

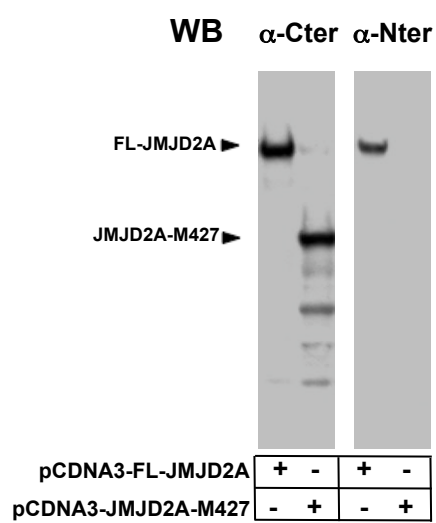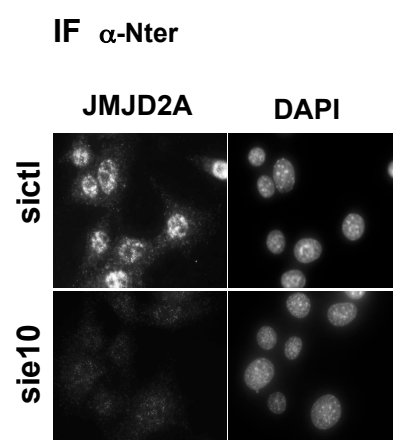

Supplement: Figure S6 — Characterisation of the antibody directed against the N-terminal part of JMJD2A. Antibodies recognizing both isoforms of JMJD2A (α-Cter antibody) or the full-length protein only (α-Nter antibody) were tested by western blot on extracts from C2C12 cells over-expressing JMJD2A full length (FL-JMJD2A) or a Nterminal deletion mutant (JMJD2A-M427). The α-Nter antibody was tested by immunofluorescence in cells transfected by a siRNA targeting exon 10 of JMJD2A (sie10) or a control siRNA (ctl). (0.31 MB PDF) [file pgen.1001390.s006.pdf]

A

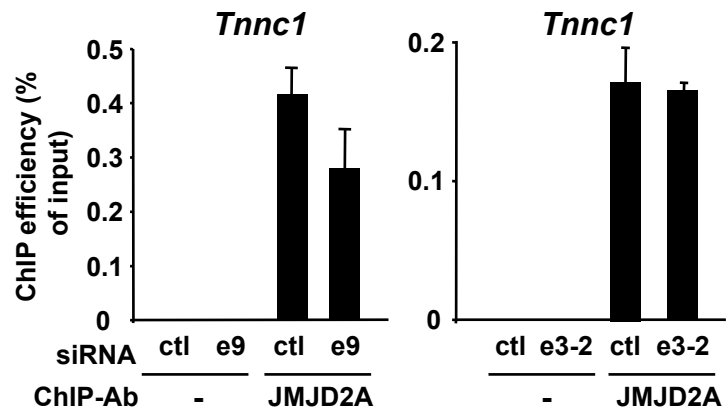

B

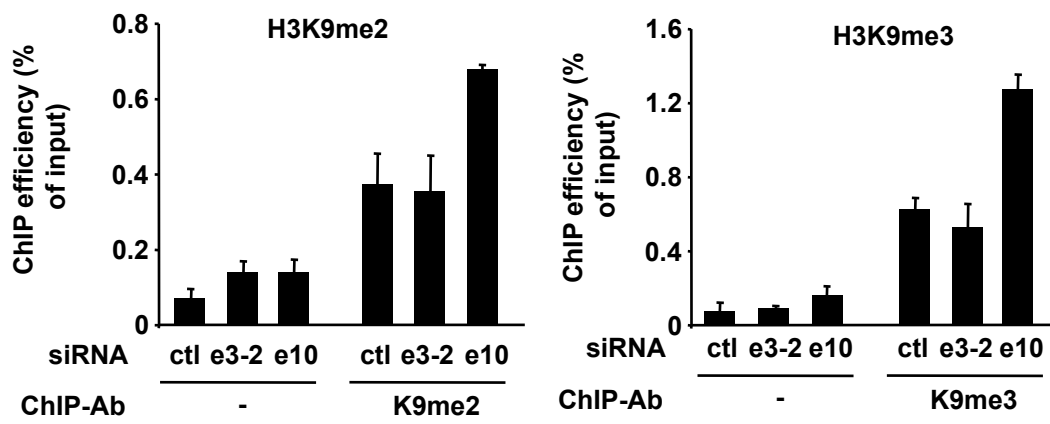

Supplement: Figure S7 — ΔN-JMJD2A targets the Tnnc1 promoter. A) C2C12 cells were transfected with siRNA targeting either exon 9 or exon 3-2, induced to differentiate and subjected to a ChIP experiment using α-CTerJMJD2A antibody. The amounts of Tnnc1 promoter in the ChIP were quantified by qPCR. A representative experiment is shown. The ChIP signal is decreased by the siRNA targeting both isoforms (sie9) but not by the siRNA targeting the full-length only (sie3-2), indicating that the Tnnc1 promoter is mainly bound by ΔN-JMJD2A. B) Same as in A, except that ChIP was performed using anti H3K9me2 (left) or anti H3K9me3 (right) antibody. (0.03 MB PDF) [file pgen.1001390.s007.pdf]

**A**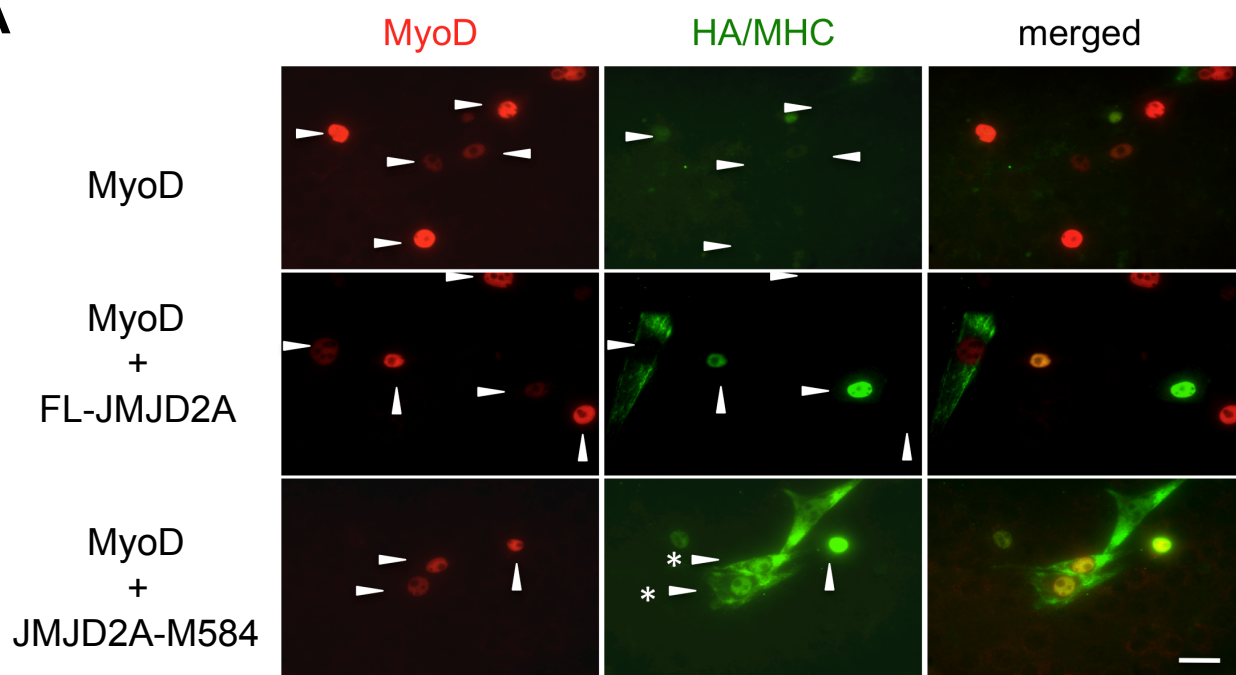**B**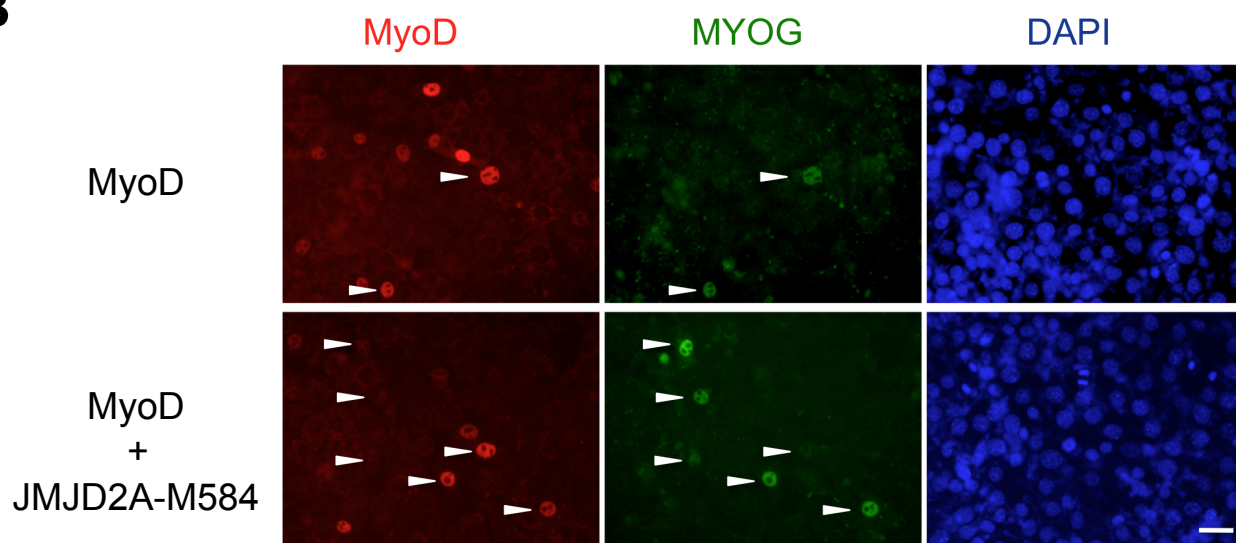

Supplement: Figure S8 — Typical images of MyoD-converted cells. A) NIH3T3 cells were transfected with MyoD alone or together with plasmids encoding FL-JMJD2A or JMJD2A-M584. Following 24 h in differentiation medium, cells were stained for MyoD in red and for both MHC and the HA epitope in green. MHC is cytoplasmic whereas HA tagged JMJD2A proteins are nuclear, allowing to discriminate between the two signals. Arrows indicate cells expressing both MHC and MyoD, and (*) cells that express both MyoD, MHC and HA. Note that one cell is stained for both MHC and HA, but does not show detectable levels of MyoD. Bar = 10 µm. B) Cells were treated as in A except they were kept in differentiation medium for 12 h and stained for MyoD in red and myogenin in green. Arrows indicate cells that express both MyoD and myogenin. Bar = 10 µm. (2.78 MB PDF) [file pgen.1001390.s008.pdf]

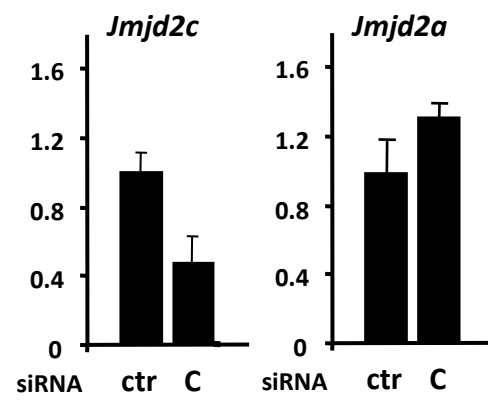

Supplement: Figure S9 — Characterisation of the siRNA targeting JMJD2C. C2C12 cells were transfected with a siRNA targeting JMJD2C (C) or a control siRNA (ctl). mRNAs were extracted, reverse-transcribed and the amounts of Jmjd2c and Jmjd2a were quantified by qPCR and calculated relative to gapdh. A representative experiment is shown. (0.03 MB PDF) [file pgen.1001390.s009.pdf]

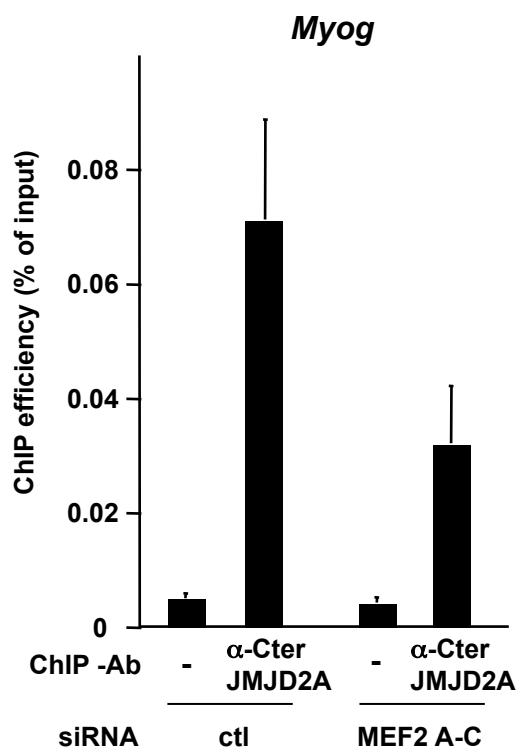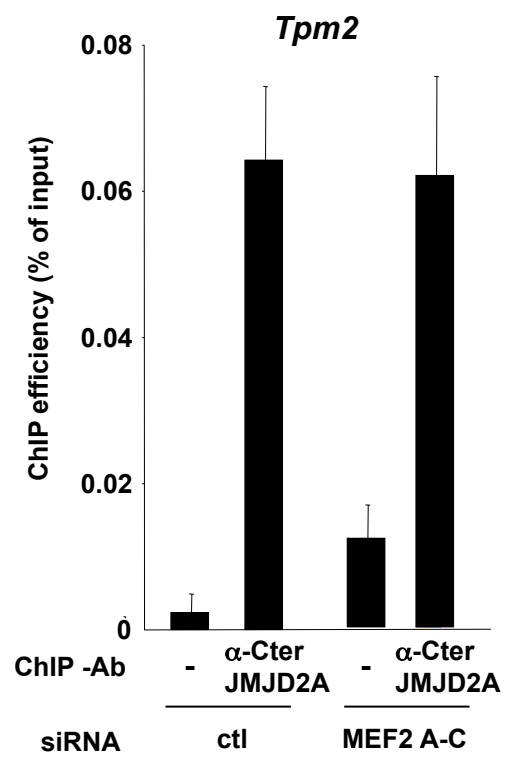

Supplement: Figure S10 — MEF2A-C expression is required for JMJD2A recruitment to the myogenin promoter. C2C12 cells were transfected with a siRNA targeting MEF2A and MEF2C or a control (ctl) siRNA. Cells were then induced to differentiate and subjected to a ChIP using the anti-CTerJMJD2A antibody. The amount of Myog (left) or Tpm2 (right) promoters in the ChIP were quantified by qPCR. A representative experiment is shown. (0.03 MB PDF) [file pgen.1001390.s010.pdf]
