# Supplementary material for: A New Isoform of the Histone Demethylase JMJD2A/KDM4A Is Required for Skeletal Muscle Differentiation
Source: PLoS Genet. 2011 Jun 2;7(6):e1001390. doi: 10.1371/journal.pgen.1001390 (PMC3107188; doi:10.1371/journal.pgen.1001390)
Supplement: Text S1 — Supplementary Materials and Methods. (0.05 MB DOC) [file pgen.1001390.s013.doc]

**Text S1: Supplemental materials and methods**

**Primers used in RT-PCR experiments** (real-time PCR)

| **Gene Symbol** | **5’-Forward primer-3’** | **5’-Reverse primer-3’** |
| --- | --- | --- |
| Actc1 | TCTCTTCCAGCCCTCTTTCA | ATGGTGGTGCCTCCAGATAG |
| Gapdh | AGGTGGTCTCCTGCGACTTC | ATGTAGGCCATGAGGTCCAC |
| Jmjd2A  Jmjd2B  Jmjd2C | GGAGCCTTGCTGAGCATCAC  TGGCTCTGCTCTTCTCCTAA  CTCCAGGCTCAAGAATGAGT | TCACTGAAGGAGCCGTCGTC  AGAATGTGCCTGCTGTCAGT  GGCTGCTGCACATTATCAAC |
| Ckm | GATTCTCACTCGCCTTCGTC | GCCCTTTTCCAGCTTCTTCT |
| Myog | CTACAGGCCTTGCTCAGCTC | AGATTGTGGGCGTCTGTAGG |
| Tnni1 | TCATGCTGAAGAGCCTGATG | AGTCCTGAAGGGCACTGAGA |
| Ttn | CCCTGACAGTGGTTGATGTG | CACTTGGGTGACCAACTCCT |

**Primers used in ChIP experiments (real-time PCR)**

| **Gene Symbol** | **5’-Forward primer-3’** | **5’-Reverse primer-3’** |
| --- | --- | --- |
| Amy2 | GTGCACATCATTGCTACTAA | ACTCGAACAGGTGGACAATA |
| Ccnb2 | ACGTTTCCGGAAGAGGAAGT | GGAGCCATCACCTAAGGACA |
| Cdc6 | TGATGAGTGACAACTAATCAG | GAGCTTTGCACTCTTCAGG |
| Cdkn1a | CGCTGCGTGACAAGAGAATA | CCTCCCCTCTGGGAATCTAA |
| Fyn | AATAGGTTCCCATGGCTGTG | GCGGAGAAGGAAGAGTTGTG |
| Gapdh | CTCTGAGCCTCCTCCAATTC | GCTACGCCATAGGTCAGGAT |
| Hfe2 | ACACAAAATCACCACCAGCA | GCAGGAACGATGAGCAAAAT |
| Id3 | CGAGGAGCCTCTTAGCCTCT | GTCTATGACACGCTGCAGGA |
| Myl6 | CCTCAGTTACCCGAAGGTCA | CTTGCCTCTCAAGCGGATAC |
| myog | GAATCACATGTAATCCACTGGA | ACGCCAACTGCTGGGTGCCA |
| Tnnc1 | GGAATGTAGCAGGAGGTGGA | TCACCGCAGCTTTGTAGATG |
| Tpm2 | ACAAGGAGAATGCCATCGAC | GCGTTAAGGCTCAACTCTGG |
| Ttn | TTGCAGCAACCACTCTTGTC | GCATGATGGGAGAGGACCTA |
| Zfp238 | AGCACAGCTGGAGGTAGCAT | CCACAGATCCGCACACACTA |

**Primers used for the quantification of FL-JMJD2A and ΔN-JMJD2A mRNA**

| **Primer pair** | **5’-Forward primer-3’** | **5’-Reverse primer-3’** |
| --- | --- | --- |
| 1 | ATGGCATTCCCTTTGACAAG | ACGGGTAGCAAAATTGGTTG |
| 2 | TGACCCTGATCTCCCCATTA | ACGGGTAGCAAAATTGGTTG |
| 3 | CAACCATGGCTTCAACTGTG | AACCTCCTCACGAACACGTC |
| 4 | TGGATTGAGTATGGCAAGCA | TGGGCAGAGTGTGGTCAATA |

**SiRNA**

The targeted sequences are as follows: sie3a GUUCGUGAGUUCCGCAAGA, sie3b GAACAUCCUACGACGAUAU, sie9 GGAAGGACAGCACGGUUAU, sie10 GUUGAGGACAGUCUUCCCU, sie11 UUGAAGAGCUGAAGAAUGU sie16 CAACAUUGCUGAAAGAAGU, sie22 AGAAGGAAGUGAAGCAAGA, siJMJD2C GCUGAAGAAGUAUGGCAUU, sictl ACUCAAACUCACGAAGGAA.

**ChIP-chips analysis**

ChIP experiments were performed as described above on C2C12 cells either in proliferative or differentiated conditions, using antibodies directed against C-terminal region (A300861A, BethylLab, 1µg/mL). Chipped DNA fragments were amplified using the WGA kit (Sigma) as recommended by the supplier. DNA labelling and hybridization on mouse promoter arrays were performed by Nimblegen. Four independent experiments were performed. Two were done by using the MM8_RefSeq_promoter array and the two others with the MM8_Deluxe_Promoter_HX1 array (Nimblegen) both covering about 20000 mouse genes. Bioinformatic analysis allowing identification of peaks was done using the Roche Nimblescan software. In order to compare the data obtained with the two distinct arrays, we further analyzed these data by restricting the promoter region to -1500 and +500 bp around the transcription start site. A promoter was recorded as bound by JMJD2A if a binding peak (p<0.01) was identified in at least three experiments.
